# Folic acid-modified antigen-trapping nanoprobes for developing in situ tumor vaccines to inhibit metastasis and recurrence of ovarian cancer

**DOI:** 10.1186/s12951-026-04537-5

**Published:** 2026-05-11

**Authors:** Xiaowen Zhong, Tao Pu, Ying Cheng, Yan Li, Qi Wang, Bin Wang

**Affiliations:** 1 Department of Anesthesiology, The First Affiliated Hospital of Chongqing Medical University, Chongqing, 400042 P. R. China; 2 Chongqing Key Laboratory of Ultrasound Molecular Imaging, Chongqing Medical University, Chongqing, 400016 P. R. China; 3 State Key Laboratory of Ultrasound in Medicine and Engineering, Chongqing Medical University, Chongqing, 400016 P. R. China

**Keywords:** *In situ* tumor vaccines, Antigen capture, Tumor microenvironment, Chemoimmunotherapy, Ovarian cancer

## Abstract

Tumor vaccines hold promise in inhibiting tumor metastasis and recurrence by stimulating the immune system to target and eliminate heterogeneous tumors. However, the development of optimal strategies for in situ tumor vaccines and the establishment of reliable methods to ensure their efficacy remain challenges. In this study, we engineered a folate-modified antigen-trapping nanoprobe (AGO@FA-lip). This nanoprobe initially acquires tumor antigens and double-stranded DNA (dsDNA) by inducing immunogenic cell death in tumor cells through the DNA-damaging agent oxaliplatin (OXA). Nano-aluminum hydroxide (nano-Al(OH)_3_) effectively captures and aggregates the released antigens, facilitating their recognition and presentation by dendritic cells. The combination of exogenous cyclic GMP-AMP (cGAMP) and dsDNA synergistically amplifies the activation of STING signaling pathways, leading to a remodeling of the tumor immune microenvironment and an enhancement of the anti-tumor immune response. In ovarian tumor models, AGO@FA-lip-mediated chemoimmunotherapy significantly inhibited tumor growth, metastasis, and recurrence, ultimately prolonging survival in mice. Transcriptomic analysis revealed extensive immune activation, particularly in pathways associated with antigen processing and presentation, T cell differentiation, and TNF inflammatory signaling. The design concept of AGO@FA-lip provides a clinically scalable strategy for developing in situ tumor vaccines to inhibit the metastasis and recurrence of ovarian cancer.

**Graphical Abstract:**

**Figure:**
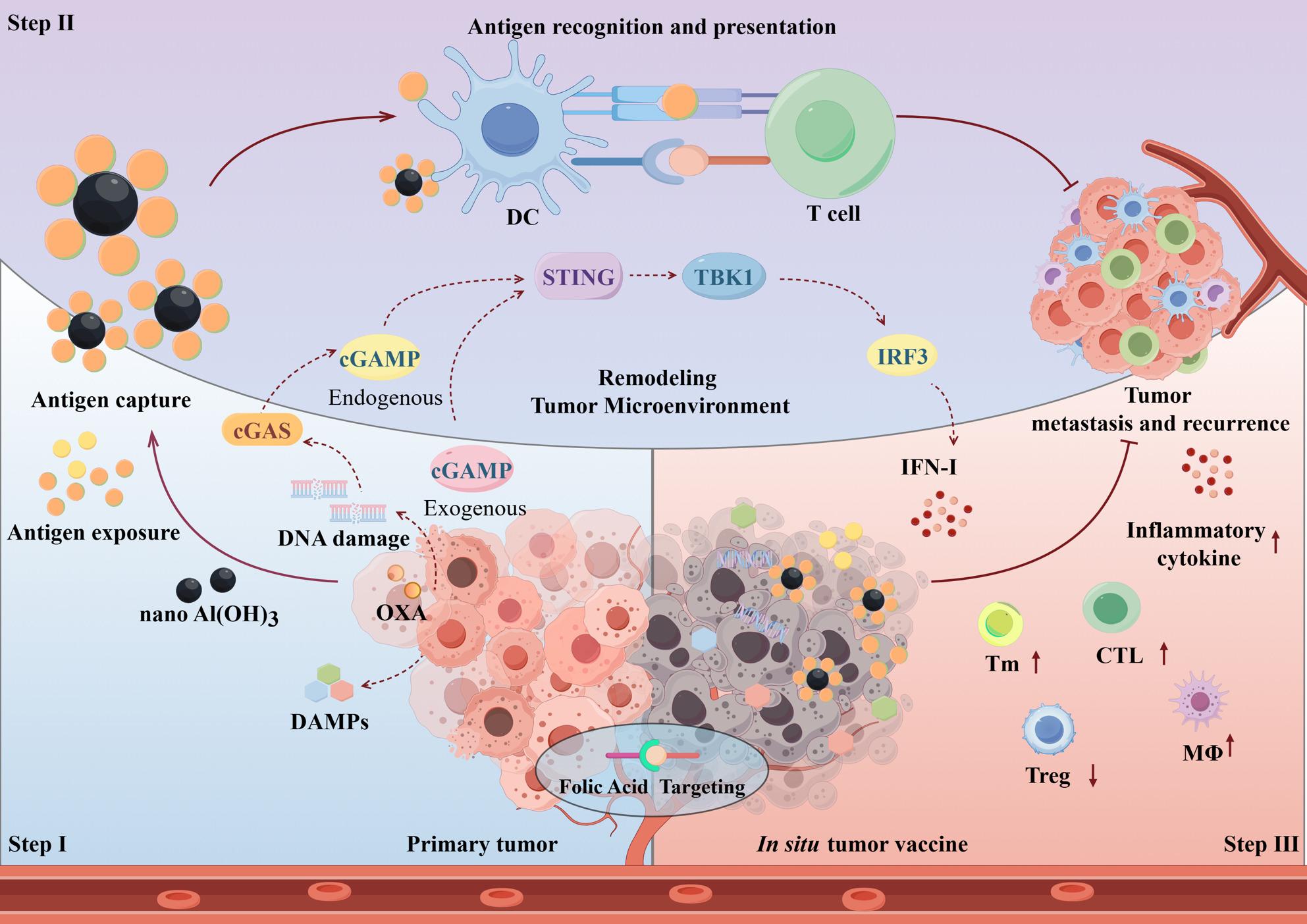
AGO@FA-lip mediated Tumor Chemoimmunotherapy and In Situ Tumor Vaccine Development (Some graphic materials were sourced from Figdraw 2.0).

**Supplementary Information:**

The online version contains supplementary material available at 10.1186/s12951-026-04537-5.

## Introduction

Ovarian cancer currently exhibits the highest mortality rate among gynecologic malignancies [1], with a recurrence rate ranging from 70% to 80% and a five-year survival rate of merely 40% [2]. The primary factors contributing to metastasis and recurrence in ovarian cancer are intraperitoneal dissemination and postoperative residual lesions [3]. Immunotherapy has emerged as a predominant cancer treatment modality, offering clinical benefits to patients with advanced-stage malignancies [4]. Tumor vaccines, which are based on tumor-specific antigens (TSA) or tumor-associated antigens (TAA), hold significant promise for tumor eradication and long-term prevention [5, 6]. Currently, sources of TSA and TAA predominantly include inactivated allogeneic or autologous tumor tissue components, tumor tissue or cell extraction complexes, tumor-associated proteins or poly-peptides, and genes encoding tumor antigens, among others [7]. However, the acquisition and preparation of these antigens are constrained by factors such as invasiveness, complexity, and high cost, and the challenge of tumor heterogeneity remains difficult to overcome. In situ tumor inactivation theoretically addresses these challenges [8, 9]. By activating the immunogenicity of inactivated tumors, it is possible to develop a personalized in situ tumor vaccine [10].

Research has indicated that strategies for tumor inactivation and the construction of in situ tumor vaccines involve the use of chemical agents, light/sound/radiation sensitizers, oncolytic viruses, and various immunostimulants [11–14]. Nevertheless, the induction of anti-tumor immunity is a multifaceted process that necessitates the coordination of diverse components within the immune system and the tumor microenvironment (TME) [15, 16]. Tumor antigens are generated through the degradation or processing of endogenous proteins released by apoptotic tumor cells. These antigens, liberated by immunogenic cell death (ICD) cells, are recognized and processed by intratumoral dendritic cells (DC), which subsequently present them to T cells via major histocompatibility complex (MHC) molecules. The MHC-antigen complex provides the initial signal required for T cell activation, while the second signal is delivered by co-stimulatory molecules expressed on mature DC. Consequently, the induction of ICD and tumor inactivation represent only the preliminary steps in the development of an in situ tumor vaccine. Tumor antigens constitute the fundamental component of tumor vaccines. Enhancing the capacity of antigen-presenting cells (APC) to more effectively recognize and present tumor antigens, thereby activating T cells, may significantly augment the efficacy of the vaccine. Long-acting adjuvants like aluminum, Freund’s complete and incomplete adjuvants, and Monophosphoryl lipid A enhance antigen uptake by APC when bound to the antigen [17]. Recent studies emphasize the potential of in situ antigen capture strategies to boost DC-mediated anti-tumor immunity [18].

The effectiveness of inactivated tumors as vaccines is influenced by tumor antigen exposure, DC antigen recognition and presentation, T lymphocyte proliferation and activation, and the immunosuppressive tumor microenvironment [19, 20]. Research indicates that patients with advanced high-grade serous carcinoma (HGSC) resistant to chemotherapy exhibit down-regulated interferon type 1 (IFN-I) gene expression and a suppressed tumor immune microenvironment [21]. Tumor cells promote immunosuppression by secreting lectins and reducing interferon synthesis [22]. Administering stimulator of the interferon genes (STING) agonists in HGSC mice increased carboplatin sensitivity and extended survival in tumor-bearing mice [23]. Activation of the cGAS-STING pathway facilitates the production of IFN-I and alleviates immunosuppression. IFN-I plays a pivotal role in remodeling the tumor immune-inflammatory microenvironment by modulating the maturation, migration, and activation of various immune cells, thereby enhancing the effectiveness of in situ tumor vaccines [24]. Nevertheless, the clinical application of pure STING agonists, such as cyclic dinucleotides, is constrained by rapid excretion, low bioavailability, and lack of specificity. Nanopharmaceutical delivery systems offer significant advantages in overcoming these challenges [25, 26]. Traditional nanocarriers have been disregarded due to their inability to evade recognition by the reticuloendothelial system [27].

**Scheme 1:**
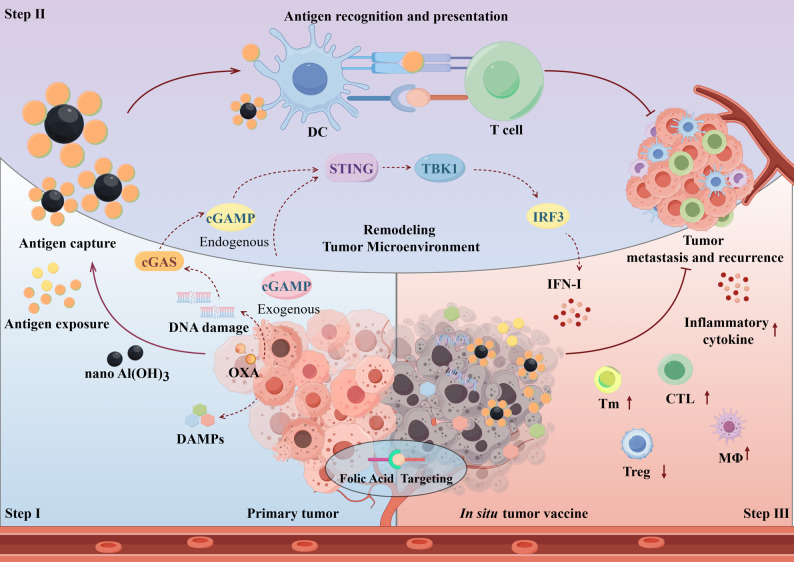
AGO@FA-lip mediated tumor chemoimmunotherapy and in situ tumor vaccine development (some graphic materials were sourced from Figdraw 2.0)

In this study, we engineered a folate-modified antigen-capture nanoprobe (AGO@FA-lip) to increase intratumoral drug concentrations and mitigate drug toxicity by specifically targeting ovarian cancer cells with high folate receptor expression [28, 29]. All raw materials used in the composition of AGO@FA-lip are FDA-approved, facilitating its potential clinical translation. Initially, AGO@FA-lip facilitated the exposure of tumor antigens and dsDNA by inducing ICD in ovarian cancer cells via OXA (Step Ⅰ). Subsequently, nano-Al(OH)_3_ efficiently captured the released tumor antigens, thereby creating optimal conditions for DC to recognize and present these antigens (Step Ⅱ). Concurrently, exogenous STING agonists, cGAMP and dsDNA, synergistically enhanced the STING signaling pathway of innate immunity, leading to increased immune cell infiltration and elevated levels of pro-inflammatory cytokines within the TME (Step Ⅲ). AGO@FA-lip not only inactivates the primary tumor but also activates specific anti-tumor immunity to eradicate metastatic tumors. The inactivated tumor acts as an in situ tumor vaccine, further preventing tumor recurrence (Scheme 1). Our findings demonstrate that AGO@FA-lip augments the anti-tumor immune response by modulating critical biological processes mentioned above, thereby effectively inhibiting tumor growth, metastasis, and recurrence in a murine model of ovarian cancer. This study presents the synthesis and functional evaluation of AGO@FA-lip, highlighting its potential as a chemoimmunotherapy agent and a strategy for in situ vaccine development against ovarian cancer.

## Experimental section

### Materials

DPPC and DSPE-PEG_2000_-FA were purchased from Ruixi Biotechnology (Xian, China). Customized services of nano-Al(OH)_3_ were provided by Ruixi Biotechnology. Cholesterol was obtained from Sigma Aldrich. cGAMP and OXA were purchased from MedChemExpress (NJ, USA). DAPI, DiI, Hoechst 33,342, and CCK-8 were obtained from Beyotime Biotechnology (Chongqing, China). Annexin V-FITC/PI was purchased from Elabscience (Wuhan, China). All antibody information for western blot (WB), flow cytometry (FCM), and immunofluorescence was annotated in Supporting Information (Table S1).

### Cell lines and animals

Mouse epithelial ovarian cancer ID8 cells were provided by Dr. Katherine Roby (University of Kansas Medical Center, USA). DC2.4 cells were acquired from the Cell Bank at the Chinese Academy of Sciences in Beijing, China. Human Umbilical Vein Endothelial Cells (HUVEC) were purchased from Fuheng Biology (Shanghai, China). ID8 cells, DC2.4 and HUV cells were cultured in a cell incubator (37 ℃, 5% CO_2_) with DMEM medium containing 10% fetal bovine serum (FBS), 1% penicillin/streptomycin. Female C57BL/6 mice, 6 to 7 weeks old and weighing 16 to 20 g, were sourced from Chongqing Medical University’s Laboratory Animal Center in China and managed in compliance with the Guidelines for the Care and Use of Laboratory Animals. All experiments involving animals received approval from the Animal Ethics Committee at Chongqing Medical University.

### Synthesis of AGO@FA-lip

#### Preparation of nano-Al(OH)_3_-cGAMP composite core

Disperse 10 mg of nano-Al(OH)_3_ in 5 mL of MES buffer (pH 6.0) and sonicate at 40 W for 5 min to ensure uniform dispersion. Dissolve 2 mg of cGAMP in 2 mL of HEPES buffer (pH 7.2), and then slowly add 2 mg of cGAMP and 10 mM MgCl₂ to the activated nano-Al(OH)_3_ solution (1 : 5 v/v). After oscillating in the dark at 4 °C for 30 min, centrifuge the mixture at 12,000 rpm for 15 min. Wash the precipitate twice with PBS to obtain the nano-Al(OH)_3_-cGAMP complex.

#### Preparation of OXA-lip

Dissolve 20 mg of lipid film (molar ratio HSPC : Chol : DSPE-PEG_2000_-FA = 65 : 35 : 5) in a chloroform-methanol mixed solvent (4 : 1 v/v) and then form a film by rotary evaporation under reduced pressure (40 °C ~ 60 °C, 120 rpm, 30 min). After vacuum drying for 4–6 h to remove organic solvents, add 2 mL of 300 mM (NH₄)₂SO₄ (pH 5.4) and hydrate at 65 °C for 30 min. Hydrated lipid films were repeatedly extruded through a 0.2 μm polycarbonate membrane to form blank liposomes. Dialyze the blank liposomes for 4 h using a dialysis bag (MWCO 8000 ~ 14000) to replace the ammonium sulfate in the external phase with PBS (pH 7.4, 4 °C). Incubate the OXA aqueous solution (5 mg/mL) with the blank liposome solution at 60 °C for 30 min. An ammonium sulfate gradient drives OXA into the liposome core. Collect the co-incubated liposomes by ultracentrifugation (5000 rpm) for 45 min, remove free OXA, and obtain oxaliplatin liposomes (OXA-lip).

#### Preparation of AGO@FA-lip

The nano-Al(OH)_3_-cGAMP complex suspension was subjected to sonication in an ice bath for 5 min (20 W, 2 s ON/5 s OFF) prior to mixing with OXA-lip. A mixture of 2 mL of the nano-Al(OH)_3_-cGAMP complex suspension (1 mg/mL in PBS) with OXA-lip at a ratio of 1:3 w/w was prepared. Rapidly freeze the mixture in liquid nitrogen for 5 min, then thaw in a 37 °C water bath for 10 min, repeating the freeze-thaw cycle 5 times. After repeatedly extruding the mixture through a 0.22 μm microfluidic membrane 5 times (pressure ≤ 0.8 bar, parameters: 65 °C, flow ratio 3 : 1), the nano-Al(OH)_3_-cGAMP-OXA@FA-lips (AGO@FA-lip) were obtained. AGO@lip, OG@FA-lip, AO@FA-lip, and AG@FA-lip were prepared by a similar method.

### Characterization of AGO@FA-lip

The morphology and structure of AGO@FA-lip were observed by scanning electron microscope (SEM, AZtecLive Ultim Max 100, Oxford Instruments) and transmission electron microscopy (TEM, Hitachi H-7600, Hitachi Ltd., Tokyo, Japan). The size, zeta potential, and PDI of the nanoprobes were measured by a dynamic laser light scattering (DLS) system on the Zetasizer Nano ZS ZEN3600 (Malvern Instruments, Malvern, UK). High-performance liquid chromatography (HPLC, Agilent 1260 Infinity II, Agilent Technologies) was employed to determine the encapsulation efficiency (EE) and loading efficiency (LE) of OXA and cGAMP.

The equations for EE and LE are as follows:

1 $$\begin{aligned}&\:\mathrm{EE\:}\left(\mathrm{\%}\right)\\&\mathrm{=}\frac{\mathrm{total\:mass\:of\:added\:drug\:-\:mass\:of\:drug\:in\:supernatant}}{\mathrm{total\:mass\:of\:added\:drug}}\times\mathrm{100\%}\end{aligned}$$

 

2 $$\begin{aligned}&\:\mathrm{LE\:(\%)}\\&\mathrm{=}\frac{\mathrm{total\:mass\:of\:added\:drug\:-\:mass\:of\:drug\:in\:supernatant}}{\mathrm{total\:mass\:of\:NPs}}\times\mathrm{100\%}\end{aligned}$$

 

The nano-Al(OH)_3_ of nanoprobes was quantitatively analyzed using energy dispersive spectroscopy (EDS, AZtecLive Ultim Max 100). Fourier transform infrared spectrometry (FT-IR, Nicolet iS50, Thermo Fisher Scientific) was used to identify the functional groups and chemical structure within AGO@FA-lip. X-ray diffraction (XRD, D8 ADVANCE, Bruker, Germany) was utilized to investigate the crystal or molecular structure inside the nanoparticles. X-ray photoelectron spectroscopy (XPS, Thermo Escalab 250Xi) analyzed the chemical states and content of aluminum and platinum in AGO@FA-lip. The drug release of AGO@FA-lip at pH 6.5, pH 7.4, glutathione solutions and hydrogen peroxide (H_2_O_2_) were tested by HPLC. The stability of the nanoparticles was evaluated in PBS, ddH_2_O, and DMEM.

### Tumor targeting

The cellular uptake of AGO@FA-lip was assessed by cellular immunofluorescence and FCM. ID8 cells or HUVECs were cultured in confocal dishes. After incubation with DiI-labeled AGO@FA-lip for different times (0.5, 1.0, 2.0, 3.0 and 4.0 h), and the cells were fixed with 4% PFA. Cellular uptake was observed by confocal laser scanning microscopy (CLSM, Dragonfly 200, UK). The uptake rate of nanoprobes by ID8 cells was measured by FCM. Tumor bodies were cultured from ID8 cells in low-adhesion wells. After incubation with AGO@FA-lip for various times, tumor bodies were collected and the nucleus was stained with propidium iodide (PI) and DAPI.

ID8 cells (100 µL, 1 × 10^6^ cells/mL) were injected subcutaneously into the right back of mice to establish subcutaneous transplanted tumors. Tumor volume was calculated as 0.5 × length × width^2^. When the tumor volume reached ~200 mm³, DiR-labeled AGO@FA-lips were injected into the tail vein of the mouse. For the establishment of the ID8 intraperitoneal metastasis tumors, 1 × 10^6^ ID8 cells were injected into the abdominal cavity of 6-week-old female C57BL/6 mice. 14 days later, these mice received an intraperitoneal injection of DiR-labeled AGO@FA-lips. Mouse fluorescence imaging was performed using an IVIS Lumina imaging system (PerkinElmer, USA) at 0.5, 1, 2, 4, 8, 12, 24, and 48 h after administration. The tumors, blood, and major organs of subcutaneous tumor-transplanted mice were collected. The Pt concentration in each component was detected by inductively coupled plasma mass spectrometry (ICP-MS, NexION300X, PerkinElmer).

### Biosafety

The biosafety of AGO@FA-lip was assessed in healthy female C57BL/6 mice. Initially, 1 mL of fresh blood was collected from 5-week-old healthy female C57BL/6 mice to prepare a 2% (v/v) RBCs suspension for the hemolysis assay. Various nanoprobes were incubated with RBCs at 37 ℃ for 2 h, and hemolysis was monitored. Phosphate-buffered saline (PBS) served as a negative control, while double-distilled water (ddH_2_O) was used as a positive control. Following centrifugation, the absorbance of free hemoglobin in the supernatant was measured at 540 nm.

3 $$\begin{aligned}&\:\mathrm{Hemolysis\:rate}\text{}\left(\mathrm{\%}\right)\\&\mathrm{=}\frac{\mathrm{A}\text{}\mathrm{NPs-A}\text{}\mathrm{PBS}}{\mathrm{A}\text{}\mathrm{water-A}\text{}\mathrm{PBS}}\times\mathrm{100\%}\end{aligned}$$

 

The cytotoxicity of AGO@FA-lips at varying concentrations (0, 5.36, 10.72, 16.08, 21.44, 26.80, 32.16 µg/mL) on HUVECs was assessed using the CCK8 assay. Additionally, the cytotoxic effects were evaluated following the co-incubation of HUVECs with AGO@FA-lips at the therapeutic concentration over different time (1, 2, 3, 6, 12, 24 h). Mouse blood and major organs, including the heart, liver, spleen, lungs, kidneys, and brain, were collected on days 1, 2, 3, 7, and 14 following intravenous administration of AGO@FA-lip. The investigation into the acute and chronic toxicological effects of AGO@FA-lip on murine health was conducted through routine hematological assessments and serum biochemical analyses, which included measurements of aspartate aminotransferase, alanine aminotransferase, creatinine, total bilirubin, creatine kinase, blood urea nitrogen, and L-lactate dehydrogenase, as well as histological examination using H&E staining. In consideration of the potential long-term toxicity associated with repeated aluminum hydroxide administration, additional samples of tumor-adjacent muscle, nerve, and brain tissue were collected from mice that exhibited the longest survival during antitumor treatment. Cellular morphology and structural integrity in these tissues were evaluated using toluidine blue staining for nerves adjacent to the tumor, Masson’s trichrome staining for muscle tissue, and H&E staining for brain tissue.

### ICD and antigen exposure

The effect of AGO@FA-lip on ID8 cell viability was assessed by CCK-8. The equivalent dose for the free drug group is OXA 5.36 µg/mL, cGAMP 2.40 µg/mL. In brief, ID8 cells were seeded in 96-well plates at a density of 6,000 cells per well. The cells were co-incubated with AGO@FA-lip for 12 h. Then, the cell viability was measured. ID8 cells were seeded in 6-well plates at a density of 3 × 10^5^ cells per well and co-incubation with nanoprobes for 12 h. Annexin V/PI was added for cell staining and flow cytometry analysis. The expression of damage-associated molecular patterns (DAMPs) was detected by cellular immunofluorescence and ELISA. AGO@FA-lips were incubated with ID8 cells in a confocal culture dish for 12 h, and the supernatant was collected. After cells were fixed with 4% PFA, PBS buffer containing 0.5% Triton X-100 and 10% goat serum was added and incubated for 60 min. Anti-calreticulin (CRT) antibodies were incubated with cells at 4 ℃ for 12 h. After PBS washing, cells were incubated with FITC-labeled goat anti-rabbit IgGH&L for 1 h. Membrane translocation of CRT in ID8 cells was observed by CLSM. Adenosine triphosphate (ATP), Heat shock protein 70 (HSP70), and High Mobility Group Protein 1 (HMGB1) in the supernatant were detected using ATP detection kit, mouse HSP70 ELISA kit, and mouse HMGB1 ELISA kit, respectively.

### Antigen capture

In order to evaluate the antigen trapping function of nano-Al(OH)_3_, TEM was used to observe the changes after nano-Al(OH)_3_ capture protein. DLS was used to measure the changes in zeta potential and hydrodynamic particle size of nano-Al(OH)_3_, while the protein content captured was estimated using the Bradford method. Sodium dodecyl sulfate-polyacrylamide gel electrophoresis (SDS-PAGE) was employed to detect the differences between proteins from lysed ID8 cells and the captured proteins. To further clarify the capture of antigens released by immunogenically dead tumor cells by nano-Al(OH)_3_, ID8 cells were incubated with GO@FA-lip for 12 h and then supernatant was collected. Nano-Al(OH)_3_ was incubated with the supernatant for 4 h to collect capture antigen. The captured proteins were detected and analyzed by an LC/MS system (QExactive^™^, Thermo). The database used for the search was the Universal Protein Mouse Proteome Reference Database (Universal Protein mouse 20190908.fast). Capture antigen peptide sequences and information are provided in the tumor antigen database TANTIGEN2.0.

### cGAS-STING pathway activation

Double-stranded DNA (dsDNA) expression in ID8 cells was detected by cellular immunofluorescence. ID8 cells were incubated with nanoprobes for 12 h and then fixed with 4% PFA. Add PBS buffer containing 0.5% Triton X-100 and 10% goat serum and incubate at room temperature for 60 min. The dsDNA-labeled antibody was incubated with the cells at 4 ℃ for 12 h. After PBS washing, cells were incubated with fluorescently labeled goat anti-rabbit IgGH&L for 1 h. DAPI marks the nucleus. dsDNA expression in ID8 cells was observed by CLSM.

The grouped ID8 cells proteins were extracted using cold RIPA buffer containing 0.1% protease inhibitors. The BCA Protein Assay Kit performs protein quantification. 30 µg of protein was loaded into each well of SDS-PAGE for electrophoresis, and then the protein was transferred to a 0.45 μm PVDF membrane and further mixed with the designated antibodies (p-STING (1 : 1000), p-TBK1 (1 : 1000), p-IRF3 (1 : 1000), STING (1 : 1000), TBK1 (1 : 1000), IRF3 (1 : 1000)) at 4 ℃ overnight. Then, an HPR conjugated goat anti-rabbit antibody was used as the secondary antibody. After washing off the secondary antibody, the blot signal was observed using a chemiluminescent imaging system (C400, Azurebiosystems, USA). IL-6, IFN-β and CXCL10 in the supernatant were tested strictly according to the instructions of mouse IL-6 ELISA kit, mouse IFN-β ELISA kit and mouse CXCL10 ELISA kit.

### DC maturation and T cell activation

Bone marrow-derived dendritic cells (BMDCs) were obtained from the bilateral femurs of 6-week-old female C57BL/6 mice. BMDCs were continuously cultured and induced for 5 days in RPMI-1640 medium containing 10% FBS, 20 ng/mL GM-CSF, and 10 ng/mL IL-4. ID8 cells were incubated with AGO@FA-lips for 12 h and the supernatant containing capture antigen was collected. After incubation of BMDCs with supernatant for 24 h, BMDCs were collected and stained for 1 h with PE anti-mouse CD11c antibody, FITC anti-mouse CD80 antibody, PC5.5 anti-mouse CD86 antibody, APC anti-mouse I-A/I-E antibody or APC anti-mouse H-2k^b^/SIINFEKL antibody. Finally, the expression levels of co-stimulatory molecules of BMDCs were detected by FCM. Cytokine IL-12 in supernatant was measured by the mouse IL-12p70 ELISA kit.

In order to observe the recognition of capture antigens by BMDCs, OVA-FITC was incubated with DiI-labeled nanoparticles to obtain labeled capture antigens. The uptake of captured antigen by BMDCs was observed by CLSM. Naive T cells were obtained from the spleen of healthy 6-week-old female C57BL/6 mice. Naive T cells were cultured in RPMI-1640 medium containing 1% penicillin/streptomycin and 10% inactivated FBS. The BMDCs incubated with captured antigens were subsequently co-cultured with naive T cells for varying durations. The captured antigens were sourced from the supernatant of inactivated tumor cells. Following a 24-h co-culture period, cells were harvested and stained with FITC-conjugated anti-mouse CD3, APC-conjugated anti-mouse CD25, and PE-conjugated anti-mouse CD69 antibodies. The expression of these markers on T cells was analyzed using FCM. Naive T cells, labeled with carboxyfluorescein succinimidyl ester (CFSE), were co-cultured for 72 h, after which T cell proliferation was assessed via FCM. After a 96-h co-culture period, the concentration of IFN-γ in the co-culture medium was quantified using ELISA.

### Antitumor effect and immune response *in vivo*

Mouse ID8 subcutaneous transplanted tumors were constructed as previously described. In vivo experiments were performed when the tumor volume reached ~200 mm^3^. Mice in each group received tail vein injection (OXA 3 mg/kg, cGAMP 1 mg/kg) on days 0, 3, and 6. Changes in tumor volume and body weight of mice were recorded every two days. Mouse sera were collected on day 7, and serum cytokine levels were measured using mouse IFN-γ ELISA kit, mouse TNF-α ELISA kit, and mouse IL-12 ELISA kit. Tumor tissue, tumor draining lymph nodes and spleen were collected on day 10. Tumor tissues collected during the survival observation period were fixed and preserved with 4% PFA, and were ultimately used to display all tumor specimens. The humane endpoints of this study include: the tumor volume of mice exceeded 1000 mm^3^, the body weight and mass decreased significantly (15% ~ 20%), the appearance of obvious endangered symptoms (obvious cyanosis of lips, obvious suppression of respiration), large-scale skin infection or abnormal central nervous response (convulsion, trembling, paralysis, tilting).

For the establishment of the ID8 intraperitoneal metastasis tumors, 1 × 10^6^ ID8 cells were injected into the abdominal cavity of 5-week-old female C57BL/6 mice. Mice in each group received intraperitoneal injection on days 0, 3, and 6. Changes in tumor volume and body weight of mice were recorded every two days. Mice were dissected on day 14, and abdominal metastases were counted, and ascites and peritoneal tumor tissue were collected. Cytokine levels in ascites were measured by the mouse IL-10, TGF-β2 or VEGF-A ELISA kit.

*FCM*. The collected tumors and spleens were prepared into single cell suspension using enzymatic lysis solution (1% collagenase, 0.5% deoxyribose, 1% hyaluronidase) or mouse tumor infiltrating lymphocyte isolation kit. ACK lysis buffer eliminates RBCs in the spleen. Dead cells were first eliminated using the Zombie NIR^™^ Fixable Viability Kit. All cells were stained after incubation with FC Block (anti-mouse CD16/32 monoclonal antibody). T lymphocytes in the tumor or spleen were labeled using PB450 anti-mouse CD45 antibody, PE anti-mouse CD3 antibody, PC5.5 anti-mouse CD4 antibody, APC anti-mouse CD8a antibody, and FITC anti-mouse CD44 antibody. DCs in spleen or tumor were labeled using PB450 anti-mouse CD45 antibody, PE anti-mouse CD11c antibody, FITC anti-mouse CD80 antibody, PC5.5 anti-mouse CD86 antibody, and APC anti-mouse I-A/I-E antibody. FCM data were acquired using CytoFLEX and analyzed with FlowJo software.

*H&E and Multiplex immunofluorescence staining (mIHC)*. Tumor tissue was fixed with 4% PFA and embedded in paraffin. Paraffinized sections were deparaffinized, hydrated, and antigen retrieved and incubated with different primary antibodies overnight at 4 ℃. PCNA monoclonal antibody and TUNEL kit were used to assess tumor cell apoptosis and proliferation. Activation of the cGAS-STING pathway in tumors was detected using p-STING rabbit antibody and dsDNA labeled antibody. The expression of DAMPs in tumors was assessed using anti-Calreticulin antibodies, anti-HMGB1 antibodies, and anti-HSP70 antibodies. Samples were then incubated with AlexaFluor^®^ 488 or Cy5-labeled secondary antibodies for 1 h. DAPI labels cell nuclei. Pannoramic P-MIDI (3DHISTECH, Hungary) was used for sample imaging. A multiplex immunofluorescence staining kit (AFIHC027, Aifang Biotech, China) was used to perform mIHC of tumor tissues and draining lymph nodes. The antibodies include CK-Pan, Anti-CD3 antibody, Anti-CD4 antibody, Anti-CD8 antibody, Anti-MHC-II antibody, Anti-GZMB antibody, Anti-F4/80 antibody, Anti-FoxP3 antibody, and Ki67. Image acquisition of mIHC slices was performed by a multi-channel fluorescence digital slide scanner (AF-KL-20-8, Aifang Biotechnology, China). Data analysis was performed on images by building custom algorithms in VISIOPHARM software (VISIOPHARM, Hoersholm, Denmark). In order to ensure the consistency of analysis results, the VISIOPHARM application program was used to set fixed and unified threshold parameters. The analysis included counts and average fluorescence intensity of CD3^+^ T cells, CD4^+^ T cells, CD8^+^ T cells, FoxP3^+^T cells, and F4/80^+^macrophages, counts and average fluorescence intensity of CD3^+^CD8^+^ T cells, CD3^+^CD4^+^ T cells, analysis of spatial distance between cells (PancK^+^ tumor cells and CD3^+^CD8^+^T cells), analysis of organizational space structure (statistics of CD3^+^CD8^+^ T cells within 50 μm of diffusion in the tumor area and statistics of CD3^+^CD8^+^ T cells within 50 μm of external diffusion).

*RNA sequencing (RNA-Seq) analysis*: The collected tumor samples from the control group and the AGO@FA-lip group were quickly rinsed with RNase-free water and then frozen in liquid nitrogen for storage. RNA sequencing was then performed by Major Biotechnology Co., Ltd. (Shanghai, China) (*n* = 3 biological replicates). Kyoto Encyclopedia of Genes and Genomes (KEGG) and Gene Ontology (GO) pathway enrichment assessment was conducted leveraging the Majorbio Cloud Platform.

### Abscopal effect and vaccine effects

*Animal model construction and treatment*. Establishment of a mouse ID8 cell subcutaneous metastasis model: Primary tumors were developed by injecting ID8 cells (100 µL, 1 × 10^6^ cells/mL) subcutaneously into the right dorsal side of mice on day 14. Distant tumors were formed by injecting ID8 cells (50 µL, 1 × 10^6^ cells/mL) into the left dorsal side of mice 7 days. Mice received intratumoral injection treatment on days 0, 3, and 6. Changes in mouse tumor volume and body weight were recorded and photographed every two days. On the 10th day, the primary tumors, distant tumors, spleens and draining lymph nodes of the mice were collected.

The vaccine effect was further evaluated through tumor cell re-challenge experiments. A mouse ID8 subcutaneous ovarian transplanted tumor model was established. When the tumor volume of mice reached ~200 mm^3^, they were treated with tail vein injection (OXA 3 mg/kg) three times. On day 14, it was observed that the volume of the subcutaneously transplanted tumor on the right back of the mice decreased significantly or even tumor disappeared. ID8 cells (100 µL, 1 × 10^6^ cells/mL) were injected under the skin on the left side of the mice’s back.

*ELISA*. The collected primary tumor tissue was washed, weighed, and ground to obtain a homogenate. The homogenate was centrifuged at 5000×g for 10 min at 2 °C ~ 8 °C, and the supernatant was collected. Intratumoral cytokine levels were determined by the mouse IFN-γ ELISA kit, the mouse CXCL10 ELISA kit, and the mouse IL-12 ELISA kit.

H&E staining and mIHC of metastases: Samples were stained and analyzed using similar methods described previously. The antibodies include CK-Pan, Anti-CD3 antibody, Anti-CD4 antibody, Anti-CD8 antibody, Anti-CD44 antibody, Anti-F4/80 antibody, Anti-FoxP3 antibody, and Ki67.

*FCM*. Collected tumors, spleens, or lymph nodes were prepared into single cell suspensions by the method described previously. T lymphocytes in the spleen or tumor were labeled using PB450 anti-mouse CD45 antibody, PE anti-mouse CD3 antibody, PC5.5 anti-mouse CD4 antibody, APC anti-mouse CD8a antibody. DCs in spleen or tumor were labeled using PB450 anti-mouse CD45 antibody, PE anti-mouse CD11c antibody, FITC anti-mouse CD80 antibody, PC5.5 anti-mouse CD86 antibody, and APC anti-mouse I-A/I-E antibody. FCM data were acquired using CytoFLEX and analyzed with FlowJo software.

### Statistical analysis

Statistical analysis was performed using GraphPad Prism 9.0 software. Data were expressed as mean ± standard deviation. One-way analysis of variance (ANOVA) with Tukey’s multiple comparisons was used to compare more than two groups. Differences between the two groups were assessed using an unpaired Student *t*-test. Kaplan-Meier survival analysis and Mantel-Cox test were used to compare the differences in overall survival of mice among groups. The Kruskal-Wallis test was a nonparametric alternative to the one-way analysis of variance test for multiple comparisons. A value of *p* < 0.05 was considered statistically significant. * *p* < 0.05, ** *p* < 0.01, *** *p* < 0.001, **** *p* < 0.0001.

## Results and discussion

### Synthesis and Characterization of AGO@FA-lip

**Fig. 1:**
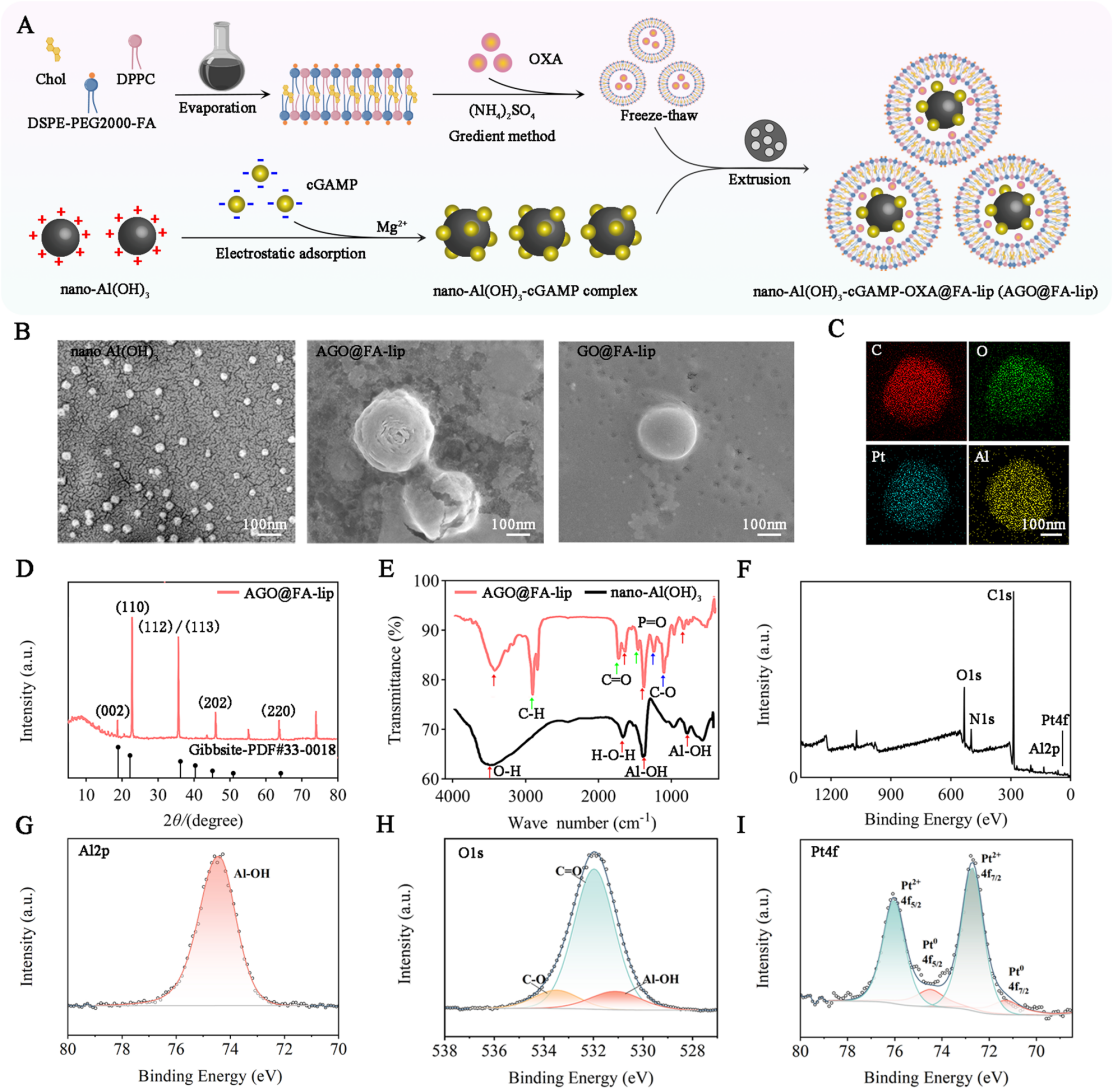
Synthesis and characterization of AGO@FA-lip. (**A**) Assembly of AGO@FA-lip. (**B**) SEM of nano-Al(OH)_3_ and AGO@FA-lip. Scale bar 100 nm. (**C**) EDS analysis of AGO@FA-lip. (**D**) XRD spectrum of AGO@FA-lip. (**E**) FT-IR spectroscopy of AGO@FA-lip. (**F**) XPS of AGO@FA-lip. G-I. Orbital peak fitting of Al, O, and Pt in AGO@FA-lip

The design and assembly process of AGO@FA-lip is illustrated in Fig. 1A. Initially, oxaliplatin liposomes (OXA-lip) were synthesized using the ammonium sulfate gradient method. Subsequently, these OXA-lip were encapsulated within AlOOH-cGAMP composite cores via a freeze-thaw extrusion technique to form AGO@FA-lip. Scanning electron microscopy (SEM) analysis revealed that AGO@FA-lip exhibited a core-shell structure, whereas GO@FA-lip, lacking nano-Al(OH)_3_, displayed a more uniform morphology (Fig. 1B). EDS analysis confirmed the presence of carbon (C), oxygen (O), platinum (Pt), and aluminum (Al), indicating the incorporation of OXA and nano-Al(OH)_3_ in the AGO@FA-lip composition (Fig. 1C, S1A). The average particle size of typical AGO@FA-lip was 216.19 ± 5.34 nm and a zeta potential of −27.51 ± 2.14 mV, with a polydispersity index of 0.11 ± 0.02 (Table S2). The loading rates of cGAMP, OXA, and nano-Al(OH)_3_ in AGO@FA-lip were 3.56 ± 0.22%, 7.96 ± 0.46%, and 14.16 ± 0.80%, respectively (Table S3). XRD results indicated that AGO@FA-lip exhibits amorphous or gibbsite peak shapes at 18.3° (002) and 0.3° (110) (Fig. 1D). The FT-IR spectrum of AGO@FA-lip showed an O-H stretching vibration peak at 3445.9 cm^− 1^, an O-H bending vibration peak at 1644.00 cm^− 1^, an Al-OH bending vibration peak at 1384.35 cm^− 1^, and an Al-O vibration peak at 769.59 cm^− 1^, indicating the presence of nano-Al(OH)_3_ (indicated by the red arrow, Fig. 1E). Compared with pure nano-Al(OH)_3_, the P-O vibrational peak at 1200 cm^− 1^ −1300 cm^− 1^ in AGO@FA-lip may originate from the cGAMP phosphate group (indicated by the blue arrow). The C-H stretching peak at 2900 cm^− 1^−2950 cm^− 1^, the C = O stretching peak at 1731.8 cm^− 1^, and the C-H bending peak at 1464.20 cm^− 1^ suggested the presence of lipid membranes (indicated by the green arrow). The characteristic peaks of Al2p and Pt4f binding energies in the XPS spectrum indicated the loading of nano-Al(OH)_3_ and OXA in AGO@FA-lip (Fig. 1F). Further analysis of the peak fitting results for the Al 2p (Fig. 1G) and O 1 s (1H) orbitals indicated that Al in AGO@FA-lip primarily exists in the form of Al(OH)_3_ (binding energy 74.0 eV − 74.5 eV). There were two main chemical states of Pt in AGO@FA-lip: zero-valent Pt (Pt^0^) and divalent Pt (Pt^2+^) (Fig. 1I). The characteristic peaks of the binding energy of Pt^0^ were located at 71.4 eV and 74.7 eV, corresponding to 4f7/2 and 4f5/2 orbitals, respectively. Pt^0^ can be attributed to partial Pt reduction during the preparation process. The characteristic peaks of the binding energy of Pt^2+^ were located at 72.1 eV and 75.4 eV, corresponding to 4f7/2 and 4f5/2 orbitals, respectively. Pt^2+^ refers to the existing form of platinum in OXA. The average particle size of AGO@FA-lip showed no significant changes in PBS, ddH_2_O, or DMEM at 4 °C over 15 days (Figure S1B).

The tumor microenvironment is characterized by hypoxia, weak acidity, and elevated levels of ROS. Consequently, our study concentrated on drug release from AGO@FA-lip under conditions of pH 5, as well as reducing or oxidizing environments. In an aqueous solution at pH 7.4, the 48-hour release efficiency of OXA from AGO@FA-lips was 19%. Under acidic conditions (pH 5), the release efficiency increased to 56% (Figure S1C). In contrast to its high sensitivity to protons (H^+^), AGO@FA-lip exhibited a slow drug release in H_2_O_2_ or glutathione solutions. In the presence of H_2_O_2_, the 48-hour release efficiency of OXA was less than 30%, while in a reducing environment, it was less than 25% (Figure S1D). It is postulated that the nano-Al(OH)_3_ present in AGO@FA-lip reacts with H^+^ in acidic conditions, thereby facilitating enhanced drug release. This mechanism may enable AGO@FA-lip to more rapidly achieve therapeutic drug concentrations within the tumor microenvironment.

### Tumor targeting and biosafety

**Fig. 2:**
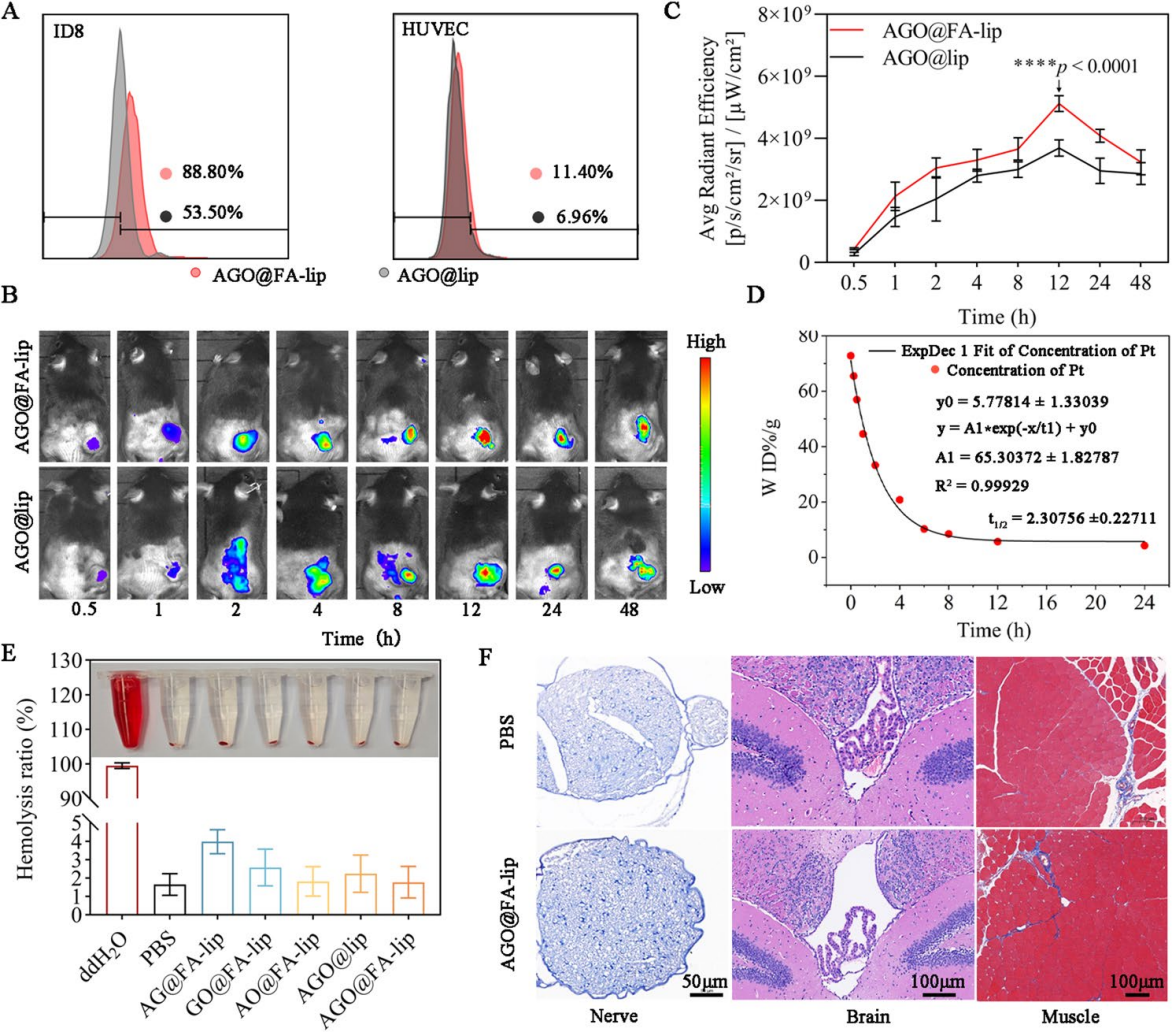
Tumor targeting, biodistribution, and biocompatibility of AGO@FA-lip. (**A**) Uptake of AGO@FA-lip by ID8 cells and HUVECs as determined by flow cytometry. (**B**) In vivo fluorescence imaging and statistical analysis of average fluorescence intensity in tumor-bearing mice within 48 h post tail vein injection of AGO@FA-lip or AGO@lip (*n* = 3). (**C**) Tumor fluorescence imaging of tumor-bearing mice at different time points following tail vein injection. (**D**) Plasma platinum (Pt) concentrations at different intervals within 24 h post tail vein injection in mice. (**E**) Hemolysis reaction after co-incubation of different nanoprobes with RBCs (*n* = 3). (**F**) Toluidine blue staining of nerves adjacent to the tumor, Masson’s trichrome staining of muscles, and H&E staining of brain tissue in subcutaneously transplanted tumor mice 60 days after repeated intravenous administration of AGO@FA-lip. * *p* < 0.05, **p < 0.01, ***p < 0.001, ****p < 0.0001

DiI-labeled AGO@FA-lips exhibited significant accumulation in the cell membrane and cytoplasm following co-incubation with ID8 cells. In contrast, the fluorescence signal of AGO@lips was weak, suggesting that tumor cells exhibited low uptake of the probe in the absence of folate modification (Figure S2A). The phagocytosis rate of AGO@FA-lips by ID8 cells was 88.80%, markedly higher than that observed in HUVECs (11.40%, Fig. 2A), demonstrating that AGO@FA-lips possess enhanced targeting capabilities for cells with elevated folate receptor expression. The IC_50_ of OXA in AGO@FA-lip for ID8 cells was determined to be 5.36 µg/mL, while the concentration of cGAMP in AGO@FA-lip was 2.40 µg/mL (Figure S2B). Subsequently, we evaluated the tumor-targeting efficacy of AGO@FA-lip in ID8 tumor-bearing mice. In vivo fluorescence imaging demonstrated that DiR-labeled AGO@FA-lips progressively accumulated in tumors over a 12-h period following intravenous administration (Fig. 2B). At the 12-h mark post-administration, the fluorescence signal intensity in the AGO@FA-lip group was significantly higher than that in the AGO@lip group (*p* < 0.0001, Fig. 2C). Folic acid modification improved AGO@FA-lip’s tumor targeting compared to AGO@lip. In contrast to the time-dependent enhancement of the fluorescence signal observed at the tumor site following intravenous administration, a sustained high fluorescence signal was detected in the abdominal region of mice with intraperitoneal metastasis tumors (Figure S3A). Within the initial 8 h post-intraperitoneal administration, no statistically significant difference in average fluorescence intensity was observed between the AGO@FA-lips group and the AGO@lips group. However, after 12 h, the AGO@FA-lips group exhibited a slightly higher average fluorescence intensity compared to the AGO@lips group (*p* < 0.05, Figure S3B). Further investigation is required to determine whether this difference is attributable to the targeted uptake of AGO@FA-lips by peritoneal tumor cells. The sustained fluorescence signal over 48 h underscores the retention of nanoprobes in the intraperitoneal tumor microenvironment. After 12 h of intravenous injection of AGO@FA-lip, tumor accumulation of Pt peaked at 8.64 ± 0.51 µg (Figure S3C), with a blood circulation half-life of 2.31 ± 0.22 h (Fig. 2D). CCK8 results indicated that the in vitro therapeutic concentration of AGO@FA-lips (5.36 µg/mL) did not significantly impact HUVEC cell viability. However, exposure to six times this concentration resulted in an 11.55 ± 3.16% reduction in HUVEC viability (Figure S4A). Furthermore, after a 12-h co-incubation with AGO@FA-lips (5.36 µg/mL), the cell viability of HUVECs was 93.06 ± 2.73%, which was substantially higher compared to that of ID8 cells (Figure S4B). No hemolytic reaction was observed after a 4-h incubation with RBCs (Fig. 2E). In vivo therapeutic doses of AGO@FA-lip (OXA 3 mg/kg) did not induce acute cardiac, hepatic, or renal dysfunction in healthy female C57BL/6 mice within 72 h (Figure S4C), and follow-up biochemical tests after 2 weeks showed no chronic organ damage. Histopathological analysis revealed no significant damage to major organs (Figure S4D). To address concerns about nano-Al(OH)_3_’s long-term toxicity, muscle, neural, and brain tissues near tumors in long-surviving mice were examined, showing no significant changes in cellular morphology or structure (Fig. 2F). These findings preliminarily suggest that AGO@FA-lip demonstrates biosafety.

### ICD and activation of the cGAS-STING pathway

**Fig. 3:**
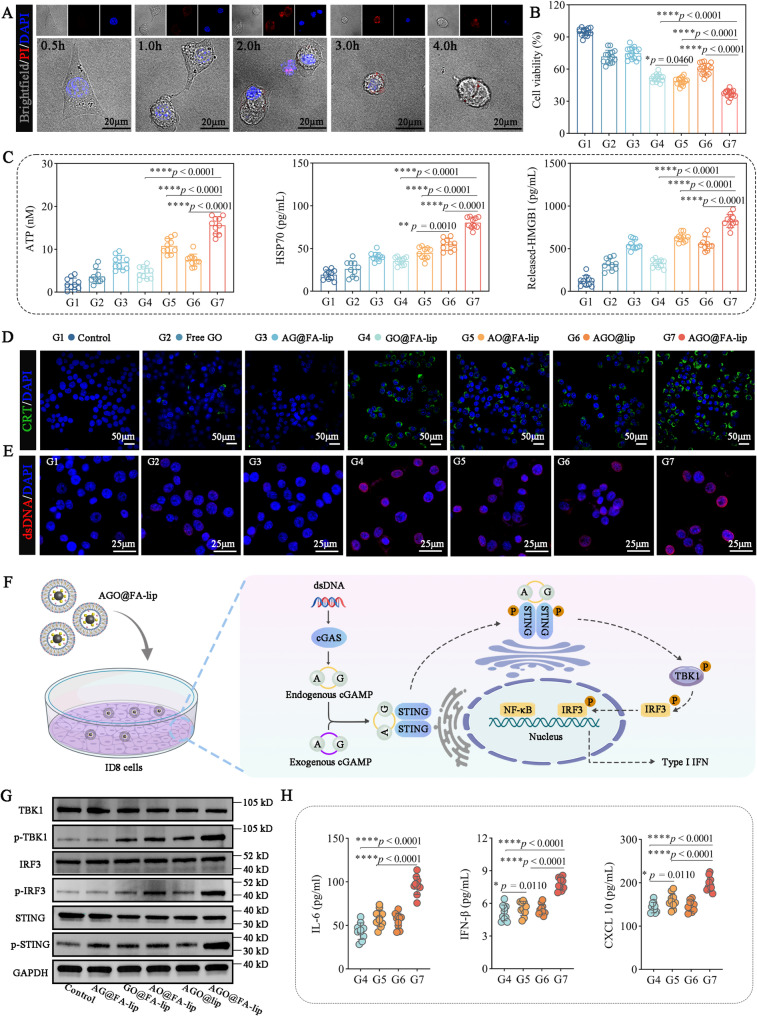
In vitro ICD and STING pathway activation. (**A**) Morphology and cell activity changes of ID8 cells after co-incubation with AGO@FA-lip for different times. Scale bar 20 μm. (**B**) CCK8 assay of ID8 cell viability after co-incubation with different nanoprobes (*n* = 10). (**C**) DAMPs (ATP, HSP70 and HMGB1) released of ID8 cells after co-incubation with different nanoparticles (*n* = 10). (**D**) CLSM observation of CRT membrane translocation in ID8 cells after co-incubation with different nanoprobes. Scale bar 50 μm. (**E**) AGO@FA-lip mediated DNA damage in ID8 cells. Scale bar 50 μm. (**F**) Schematic diagram of tumor cells inactivated by AGO@FA-lip and synergistically activating the STING pathway. (**g**) Western blot analysis of STING, TBK1, IRF3, and their phosphorylation levels. (**H**) ELISA detection of the secretion of downstream cytokines IL-6, IFN-β, and CXCL 10 after STING pathway activation (*n* = 10). * *p* < 0.05, ** p < 0.01, *** p < 0.001, **** p < 0.0001

To assess the cytotoxicity and induction of ICD by AGO@FA-lip in tumor cells, we initially examined the morphological changes in tumor cells and tumor spheroids following co-incubation with AGO@FA-lip. With prolonged co-cultivation, ID8 cells progressively lost their morphology, while the red fluorescent signal of PI, a marker for cell apoptosis, increased within the nucleus (Fig. 3A). This observation indicates that AGO@FA-lip can rapidly induce apoptosis in targeted cancer cells. The cell survival rate in the AGO@FA-lip group was 37.21 ± 3.24% lower than that in the AGO@lip group (*p* < 0.0001, Fig. 3B), suggesting that folic acid modification enhances the cytotoxicity of the nanoprobes against tumor cells. Upon intracellular hydrolytic activation, Pt^2+^ released from OXA bind to the N7 position of guanine in DNA to form Pt-DNA adducts. These intrastrand crosslinks can hinder the replication and transcription of tumor cell DNA, thereby inducing apoptosis [30]. The apoptosis rates of ID8 cells in the AG@FA-lip and AGO@FA-lip groups were 16.78 ± 3.06% and 66.44 ± 4.24%, respectively (Figure S5), suggesting that the inclusion of OXA enhances the tumor cell-killing efficacy of AGO@FA-lip.

Anthracyclines induce cancer cell death by activating the immune system through a process known as ICD, which is characterized by the release of membrane-bound soluble factors and the enhancement of immune cell function [31]. We observed increased secretion of damage-associated molecular patterns (DAMPs) in tumor cell supernatants following co-culture with nanoprobes. Specifically, the concentrations of ATP, HSP70, and HMGB1 in the AGO@FA-lip group were significantly elevated compared to the OXA-free nanoprobe groups (*p* < 0.0001, Fig. 3C). Additionally, strong expression of calreticulin (CRT) was detected in the cytoplasm and cell membrane of ID8 cells treated with AGO@FA-lip, whereas the fluorescence signal in the AG@FA-lip group lacking OXA was nearly negligible (Fig. 3D). These findings indicate that AGO@FA-lip induces cancer cell death through immunogenic mechanisms.

In addition, a more pronounced degree of DNA damage was observed in ID8 cells following co-incubation with AGO@FA-lip (Fig. 3E). Upon activation of the cGAS-STING pathway by cytoplasmic DNA in tumor cells, the STING protein relocates from the endoplasmic reticulum to the Golgi apparatus, subsequently activating downstream transcription factors IRF3 and NF-κB. These transcription factors are responsible for inducing the production of type I interferons and pro-inflammatory cytokines, respectively [32]. Exogenous cGAMP, delivered via AGO@FA-lip, acts as a STING activator and may synergistically interact with dsDNA to further amplify the signaling of this pathway [33]. To evaluate this hypothesis (Fig. 3F), we investigated the effects of AGO@FA-lip treatment on the cGAS-STING pathway in ID8 cells. Western blot analysis demonstrated that the phosphorylation levels of STING, TBK1, and IRF3 were significantly elevated in the AGO@FA-lip group compared to the AO@FA-lip and AG@FA-lip groups (Fig. 3G). Additionally, the concentrations of IL-6, IFN-β, and CXCL10 in the cell supernatant of the AGO@FA-lip group were markedly increased (*p* < 0.0001, Fig. 3H), suggesting an enhanced secretion of downstream cytokines associated with the cGAS-STING pathway in tumor cells. The results indicate that the simultaneous administration of DNA-damaging agents and STING agonists to tumor cells through nano-Al(OH)_3_ can enhance the activation of the cGAS-STING pathway more effectively. The ICD of tumor cells and the type I interferon response elicited by AGO@FA-lip contribute positively to the initiation of subsequent immune cascade reactions, thereby establishing critical prerequisites for the development of in situ tumor vaccines.

### Tumor antigen exposure and capture

**Fig. 4:**
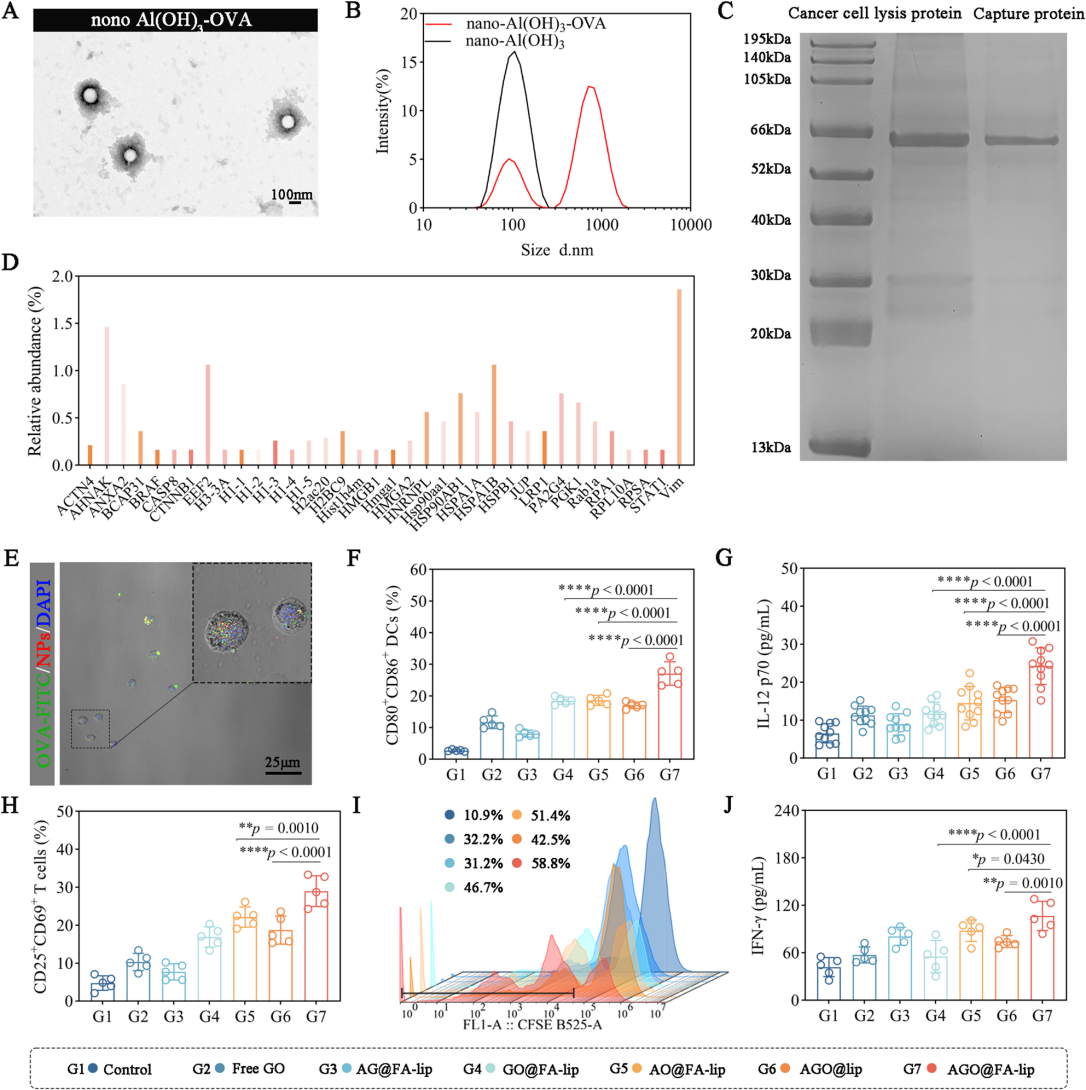
Antigen Capture and In Vitro Immune Activation of AGO@FA-lip. (**A**) Transmission electron microscope image of nano-Al(OH)_3_-OVA. Scale bar 100 nm. (**B**) Particle size changes of nano-Al(OH)_3_ after protein adsorption. (**C**) SDS-PAGE protein analysis of cancer cell lysate protein and capture protein. (**D**) The relative abundance of antigens captured by AGO@FA-lip. (**E**) CLSM observation of DCs uptake of captured OVA-FITC antigens. Scale bar 25 μm. (**F**) Statistical analysis of CD80^+^CD86^+^ DCs detected by flow cytometry (*n* = 5). (**G**) ELISA detection of cytokines IL-12p70 secreted by DCs (*n* = 10). (**H**) Activation of naïve T cells after co-culture with antigen-loaded mature DCs for 24 h. (**I**) T cell proliferation after co-culture with antigen-loaded mature DCs for 72 h. (**J**) The positive rate of IFN-γ^+^ T cells after co-culturing with antigen-loaded mature dendritic cells for 96 h. * *p* < 0.05, ** p < 0.01, *** p < 0.001, **** p < 0.0001

 To assess the antigen capture capability of AGO@FA-lip, we initially examined the protein adsorption properties of nano-Al(OH)_3_. TEM revealed significant deposition and adhesion on the surface of nano-Al(OH)_3_ following incubation with ovalbumin (OVA) (Fig. 4A). Following the adsorption of OVA, the zeta potential of nano-Al(OH)_3_ transitioned to a negative value (−4.30 ± 0.77 mV, Figure S6A), and the average particle size increased to the micron scale (1056.13 ± 27.52 nm, Fig. 4B). The capture efficiency of tumor lytic proteins was quantified using the Bradford assay, revealing a reduction in the protein capture capacity of nano-Al(OH)_3_ when coated with a lipid film (Figure S6B). Specifically, the protein capture capacities of nano-Al(OH)_3_ and AGO@FA-lip were determined to be 593.41 µg/mL and 248.13 µg/mL, respectively. It was observed that Al(OH)_3_ nanoparticles lack the specificity required for capturing protein antigens. In the serum co-incubation experiment, distinct differences were noted in the protein profiles captured by nano-Al(OH)_3_ and AGO@FA-lip (Figure S6C), suggesting that AGO@FA-lip exhibits adsorption to non-tumor proteins. Further analysis via SDS-PAGE revealed that the protein profile of the captured tumor proteins differed from that of the ID8 cell lysate proteins (Fig. 4C). Subsequent mass spectrometry and label-free quantitative analysis of the captured tumor cell lysate proteins identified a total of 1865 major proteins containing specific peptides, including 36 known TAAs (Table S4, Fig. 4D). The mechanism of antigen adsorption by nano-Al(OH)_3_ primarily involves electrostatic adsorption, ligand exchange, and hydrophobic interactions [34]. These findings establish a foundation for AGO@FA-lip-mediated tumor antigen capture.

### DC maturation and T cell activation

To assess the recognition of nanoprobe-captured antigens by DCs, we initially examined the uptake of OVA by DCs. CLSM revealed that OVA captured by the nanoprobe accumulated in the cell membrane and cytoplasm of DCs (Fig. 4E). The supernatant containing the captured antigen was collected following a 12-h incubation of the nanoprobes with ID8 cells. FCM analysis demonstrated that, after co-incubation with the captured antigen, the AGO@FA-lip group exhibited the highest proportion of CD80^+^CD86^+^ DCs (Fig. 4F, S7A). Furthermore, the concentration of interleukin-12 (IL-12) in the supernatant of DCs from the AGO@FA-lip group was significantly higher than that in the GO@FA-lip and free drug groups (*p* < 0.0001, Fig. 4G). In aluminum-based vaccines, antigens can be extensively aggregated either on the surface or within the aluminum adjuvant while preserving stable physical and chemical properties [35]. Soluble antigens can be transformed into particulate forms through conjugation with aluminum-based adjuvants [36]. This transformation creates favorable conditions for DCs to recognize and process these antigens. Compared to GO@FA-lip, AGO@FA-lip facilitated the maturation of a greater number of DCs, potentially due to the adsorption of antigens by nano-Al(OH)_3_.

To evaluate whether tumor antigens internalized by DCs can activate T cells, we performed co-culture experiments involving DCs with captured antigens and naive T cells. FCM analysis conducted after 24 h of co-culture revealed varying levels of T cell activation in the nanoparticle-treated groups. The AGO@FA-lip group exhibited the highest proportion of CD25^+^CD69^+^ T cells (*p* < 0.01, Figs. 4H, S7B). T cell proliferation was most pronounced in the AGO@FA-lip group after 72 h of co-culture (Fig. 4I). Furthermore, after 96 h of co-culture, the IFN-γ concentration in the cell supernatant of the AGO@FA-lip group was 1.32, 1.93, 1.21, and 1.43 times higher than that of the AG@FA-lip, GO@FA-lip, AO@FA-lip, and AGO@lip groups, respectively (Fig. 4J).

The antigens present in the vaccine initiate activation of either CD8^+^ T cells or CD4^+^ T cells, contingent upon the epitope length. Epitopes comprising amino acid sequences of 8–12 residues typically associate with MHC class I molecules and stimulate CD8^+^ T cells, whereas epitopes with amino acid sequences of 13–25 residues generally bind to MHC class II molecules, thereby activating CD4^+^ T cells. APCs process longer epitopes to generate both MHC class I and II antigens [37, 38]. Pro-inflammatory cytokines, such as IL-12, secreted by mature DCs, provide essential signals for T cell differentiation and survival [39]. In conjunction with previous analytical results, it is evident that the activation level of T cells is associated with the immunogenic cell death of ID8 cells and the maturation of DCs. Following the induction of ID8 cells by AGO@FA-lip, which resulted in the release of elevated levels of DAMPs and dsDNA, DCs exhibited significant expression of CD80 and CD86, along with enhanced secretion of IL-12. When co-cultured with naive T cells, DCs may provide two critical signals necessary for T cell activation: MHC-antigen complexes and costimulatory molecules. While GO@FA-lip demonstrated efficacy in inducing ICD and activating the cGAS-STING pathway in ID8 cells, it was less effective than AGO@FA-lip in promoting DC maturation and T cell activation. This disparity suggests that the capture of tumor antigens by nano-Al(OH)_3_ could be a critical factor in enhancing anti-tumor immune responses. AG@FA-lip’s inability to effectively kill tumor cells is attributed to the absence of OXA, while AO@FA-lip, lacking cGAMP, showed inadequate activation of the cGAS-STING pathway in tumor cells. Significant advancements in cancer therapy have been achieved through the integration of multiple regulatory strategies by nanosystems [40–42]. These findings imply that the concurrent delivery of DNA-damaging agents and STING agonists to tumors via nano-Al(OH)_3_ represents a more effective strategy. Furthermore, folic acid modification enables this drug delivery system to specifically target tumor cells, thereby increasing intratumoral concentrations of cGAMP and OXA while minimizing drug toxicity.

### *In vivo* antitumor effects

**Fig. 5:**
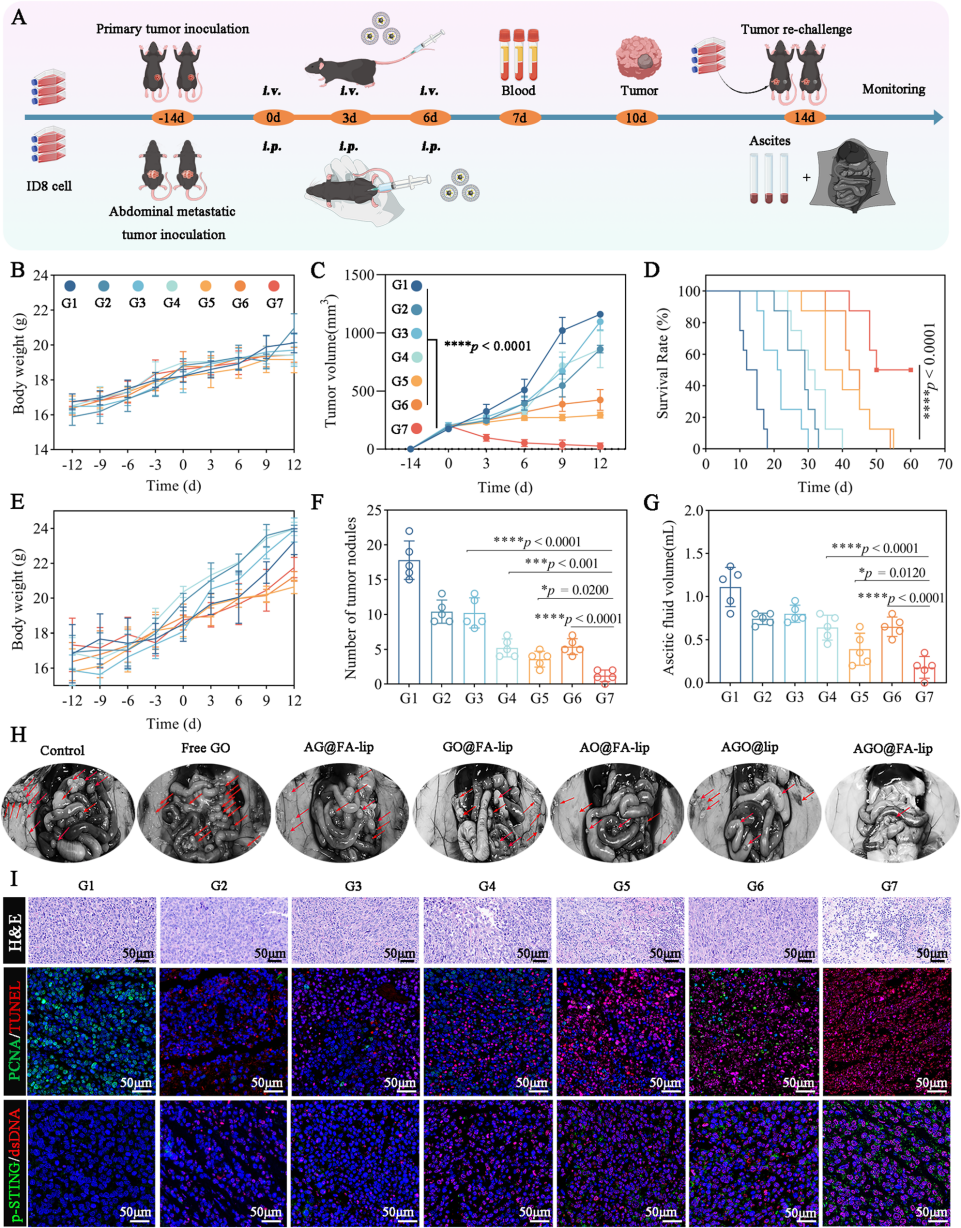
In vivo antitumor efficacy of AGO@FA-lip. (**A**) Schematic diagram of treatment for subcutaneously transplanted tumors and intraperitoneal metastasis tumors in vivo. (**B**) Body weight of mice with subcutaneous transplanted tumors (*n* = 5). (**C**) Tumor volume changes in mice with subcutaneously transplanted tumors after treatment (*n* = 5). (**D**) Survival statistics of mice with subcutaneously transplanted tumors after treatment (*n* = 5). (**E**) Body weight of mice with intraperitoneal metastasis tumors (*n* = 5). (**F**) Statistical analysis of the number of tumor nodules in mice with intraperitoneal metastasis tumors after treatment (*n* = 5). (**G**) Statistical analysis of the ascitic fluid volume in mice with intraperitoneal metastasis tumors after treatment (*n* = 5). (**H**) Anatomical diagram of mice with intraperitoneal metastasis tumors on the 21 st day of treatment. (**I**) H&E staining, PCNA/TUNEL, and dsDNA/p-STING immunofluorescence images of subcutaneously transplanted tumors. Scale bar 50 μm. * *p* < 0.05, ** p < 0.01, *** p < 0.001, **** p < 0.0001

Subsequently, we assessed the anti-tumor efficacy of AGO@FA-lip using a mouse model with subcutaneous transplantation of ID8 cells. The specific treatment protocols and evaluation methods are depicted in Fig. 5A. Throughout the treatment period, no significant reduction in body weight was observed in any of the tumor-bearing mice (Fig. 5B). By the 12th day of treatment, the AGO@FA-lip group exhibited a significantly greater inhibition of tumor growth compared to the other groups. The average tumor volumes in the control, Free GO, AG@FA-lip, GO@FA-lip, AO@FA-lip, and AGO@lip groups were 39.69, 30.18, 26.10, 18.81, 11.37, and 16.45 times greater than that of the AGO@FA-lip group, respectively (*p* < 0.0001, Fig. 5C). Furthermore, mice in the AGO@FA-lip group demonstrated prolonged survival following the reduction of tumor burden (Fig. 5D). Compared to the GO@FA-lip group, the AGO@FA-lip group exhibited smaller average tumor volumes and extended survival times, potentially attributable to the antigen capture and adjuvant effects of nano-Al(OH)_3_ [17, 35, 36, 43]. In the evaluation of Response Evaluation Criteria in Solid Tumors (RECIST) results (Figure S8A), the AGO@FA-lip treatment group of mice exhibited the highest proportion of complete response (CR, defined as the complete disappearance of tumor lesions with no new lesions appearing) at 40%, and partial response (PR, defined as a reduction in the diameter of tumor lesions by ≥ 30% compared to the baseline volume) at 60%. Conversely, the remaining groups demonstrated 100% disease progression (PD, defined as an increase in the sum of the maximum diameters of the target tumor lesions by at least 20%, or the appearance of new lesions), or the appearance of new lesions, or stable disease (SD, defined as a decrease in the sum of the maximum diameters of tumor lesions by less than 30% or an increase by less than 20%).

In the context of intraperitoneal chemotherapy for patients with advanced ovarian cancer, chemotherapeutic agents are administered directly into the abdominal cavity to elevate local drug concentrations and thereby enhance therapeutic efficacy. Given the intraperitoneal spread of ovarian cancer, we assessed the anticancer efficacy of intraperitoneal AGO@FA-lip injections in metastatic tumors. Tumor-bearing mice exhibited varying weight increases due to ascites and tumor growth (Fig. 5E). The AGO@FA-lip group showed the fewest tumor nodules (Fig. 5F) and the least ascites volume (Fig. 5G), while the control group had the most tumor nodules, highlighted by red arrows in Fig. 5H. The significant tumor suppression observed with AGO@FA-lip in both intravenous and intraperitoneal treatments supports its potential for clinical application.

To further evaluate AGO@FA-lip’s anti-tumor effects, we conducted immunohistochemical analysis. In the H&E staining of tumor tissues from subcutaneous transplanted tumors and intraperitoneal metastasis tumors, the OXA-containing nanoprobe groups exhibited more pronounced tissue destruction and abnormal cellular structures. Notably, the AGO@FA-lip group demonstrated the most significant tumor cell lysis or swelling, as well as nuclear fragmentation (Fig. 5I, S8B). These findings emphasize the vital contribution of OXA to the development of antigen-trapping nanoprobes. Tumors treated with AGO@FA-lip experienced more extensive apoptosis compared to those in the AGO@lip group (Fig. 5I), highlighting the significance of folic acid modification in enhancing the efficacy of antigen-trapping nanoprobes [28]. Immunofluorescence staining revealed DNA damage in tumor tissues treated with AGO@FA-lip (Fig. 5I), which is attributed to OXA’s direct targeting of DNA damage in tumor cells [30]. Concurrently, there was a marked expression of p-STING, indicating activation of the cGAS-STING pathway within the tumor. The fluorescence intensity of p-STING was significantly elevated in the AGO@FA-lip group relative to the AO@FA-lip and AG@FA-lip groups (*p* < 0.0001, Figure S8C).

The dsDNA of cancer cells is transported into the cytoplasm of BATF3-dependent DCs via tumor-derived exosomes, which subsequently activate the STING-dependent production of interferons, thereby enhancing anti-tumor immune responses [44–46]. Consequently, we hypothesized that the superior anti-tumor efficacy of AGO@FA-lip compared to AO@FA-lip and AG@FA-lip is associated with the DNA damage induced by OXA and the heightened activation of the cGAS-STING pathway [47, 48]. This finding further corroborates our hypothesis presented in the in vitro study section, suggesting that the concurrent delivery of DNA-damaging agents and STING agonists to tumors via nano-Al(OH)_3_ represents a more effective strategy.

### Remoulding the tumor microenvironment by AGO@FA-lip

**Fig. 6:**
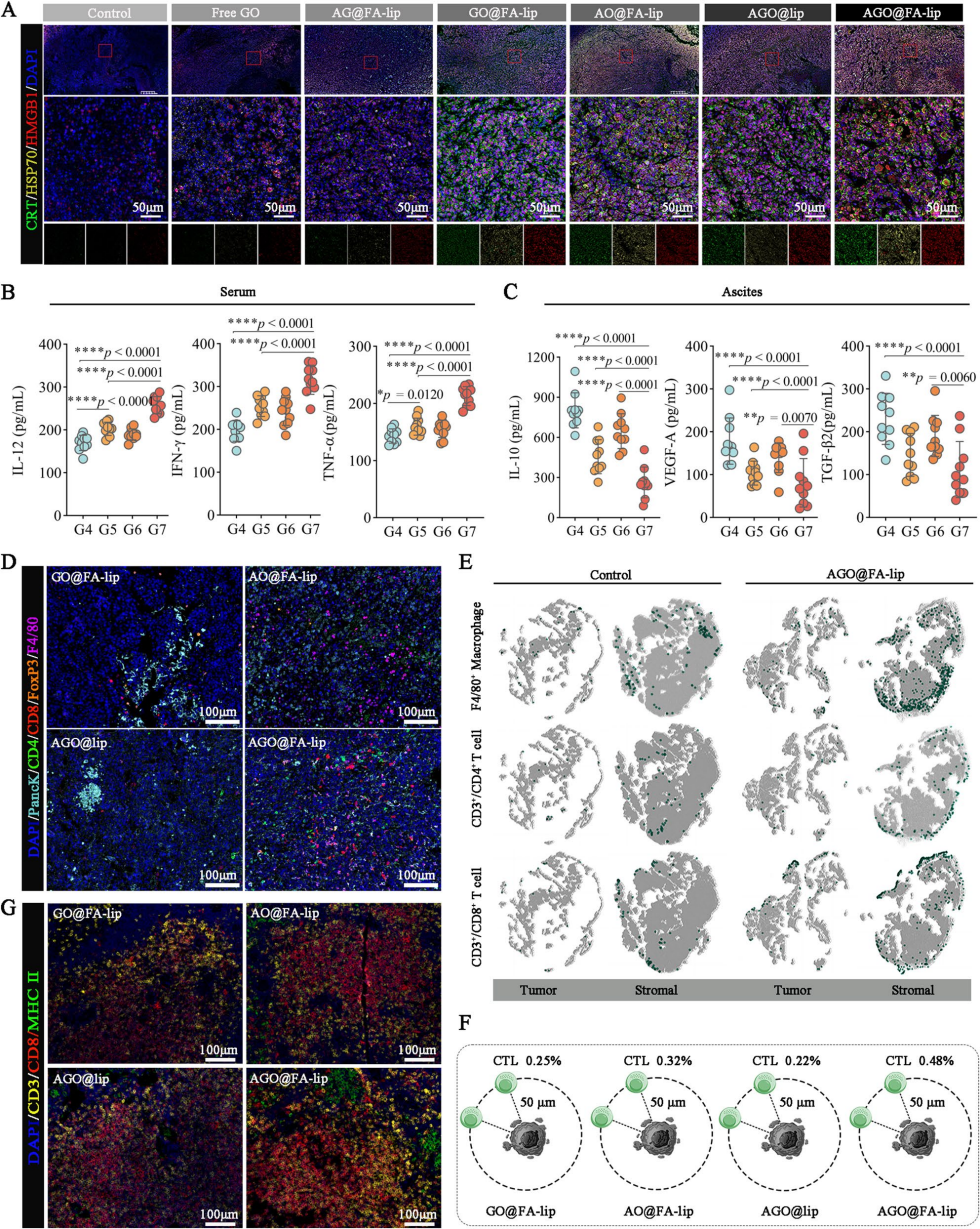
In vivo tumor immune microenvironment remodeling mediated by AGO@FA-lip. (**A**) mIHC of intratumoral DAMPs (CRT, HSP70 and HMGB1) expression after AGO@FA-lip treatment. Scale bar 50 μm. (**B**) IL-12, IFN-γ and TNF-α in the serum of mice with subcutaneously transplanted tumors on the 7th day of treatment (*n* = 10). (**C**) IL-10, VEGF-A and TGF-β2 in ascites of mice with intraperitoneal metastasis tumors on day 14 of treatment (*n* = 10). (**D**) mIHC of CD4^+^ T cells, CD8^+^ T cells, F4/80^+^ macrophages and FoxP3^+^ T cells of subcutaneously transplanted tumors after treatment. Scale bar 100 μm. (**E**) Organizational spatial structure analysis of CD3^+^CD8^+^ T cells, CD3^+^CD4^+^ T cells, and F4/80^+^ macrophages of subcutaneously transplanted tumors after treatment. (**F**) Intercellular space distance analysis of CD3^+^CD8^+^ T cells and tumor cells. The distance between cells is 50 μm. (**G**) mIHC of CD3^+^ T cells, CD8^+^ T cells and MHC Ⅱ^+^ DCs of tumor-draining lymph nodes. Scale bar 100 μm. * *p* < 0.05, **p < 0.01, ***p < 0.001, ****p < 0.0001

 To assess whether AGO@FA-lip can induce ICD in tumor cells in vivo, we examined the intratumoral expression of DAMPs. Multiplex immunohistochemistry (mIHC) analysis revealed that intratumoral cells in the AGO@FA-lip treatment group exhibited elevated levels of extracellular HMGB1 secretion, CRT membrane translocation, and HSP70 expression (Fig. 6A). The role of CRT and HSP70 as ‘eat me’ signals assists DCs in the detection and phagocytosis of cancer cells [49, 50]. HMGB1 serves as a critical mediator at the intersection of innate and adaptive immunity [51]. It is hypothesized that AGO@FA-lip may also promote the exposure of other unexamined TAAs and potentially induce the expression of neoantigens within tumor cells [52]. Subsequently, we analyzed pro-inflammatory cytokines in serum within a subcutaneous tumor model and measured immunosuppressive cytokines in ascites in an abdominal metastasis model. On the seventh day of treatment, mice with subcutaneous transplanted tumors, treated via intravenous administration of AGO@FA-lip, exhibited significantly elevated serum levels of IL-12, TNF-α, and IFN-γ compared to other treatment groups. This suggests that anti-tumor immunity was activated early in the treatment (*p* < 0.0001, Fig. 6B, S9A). The findings indicate that AGO@FA-lip is capable of inducing ICD in tumor cells in vivo and broadly activating anti-tumor immunity, which is essential for the development of in situ tumor vaccines following tumor inactivation [53, 54]. Intravenous administration primarily influences the systemic circulation, where serum cytokine levels, such as IL-12 and IFN-γ, can serve as biomarkers of systemic Th1-mediated anti-tumor immunity, thereby reflecting the immune activation induced by AGO@FA-lip at distal sites [55]. Conversely, intraperitoneal administration directly impacts the local tumor microenvironment. Analysis of ascites reveals the enrichment of immunosuppressive factors (TGF-β2, VEGF-A), driven by tumor-associated immune cells (Tregs, M2-TAMs), which significantly contribute to immune evasion in advanced tumors [56]. The concentrations of IL-10, TGF-β2, and VEGF-A in the ascites of mice treated with intraperitoneal injection of AGO@FA-lip were reduced (Fig. 6C, S9B), suggesting that AGO@FA-lip inhibits abdominal tumor growth and induces alterations in the abdominal immune microenvironment.

 To further elucidate the impact of AGO@FA-lip on the tumor immune microenvironment, we examined the infiltration of intratumoral immune cells. Post-treatment with AGO@FA-lip, there was a notable increase in the infiltration of intratumoral CD3^+^CD4^+^ T cells, CD3^+^CD8^+^ T cells, and F4/80^+^ macrophages, while the number of FoxP3^+^ Treg cells decreased (Fig. 6D, S10). The ID8 cell line is derived from the spontaneous transformation of mouse ovarian surface epithelium and serves as an epithelial-derived tumor model. PanCK, a specific marker for epithelial cells, targets cytokeratin intermediate filaments and is extensively utilized to label tumor cells of epithelial origin, such as those found in ovarian cancer. In the mIHC analysis of ID8 subcutaneous transplanted tumors, PanCK staining effectively delineated tumor boundaries. Analysis of the organizational spatial structure revealed a significant increase in CD3^+^CD8^+^ T cells, CD3^+^CD4^+^ T cells, and F4/80^+^ macrophages within the tumor stroma (Fig. 6E). Furthermore, spatial distance analysis indicated that AGO@FA-lip-treated tumor cells exhibited the highest proportion of CD3^+^CD8^+^ T cells within the 0–50 μm range (Fig. 6F).

 In the AGO@FA-lip group, the number of CD8^+^ T cells was found to be 12.65, 8.31, 5.57, 2.95, 1.33, and 2.83 times greater than those in the control group, Free GO group, AG@FA-lip group, GO@FA-lip group, AO@FA-lip group, and AGO@lip group, respectively (Table S5). Moreover, the levels of IFN-γ and TNF-α within the tumor were higher in the AGO@FA-lip group compared to the AO@FA-lip and AG@FA-lip groups (Figure S11A). This observation further supports the notion that the integration of STING agonists with DNA-damaging agents can potentiate STING activation and remodel the immunosuppressive microenvironment [54, 57]. Compared to GO@FA-lip, AGO@FA-lip demonstrated superior efficacy in inhibiting tumor growth and enhancing the intratumoral infiltration of cytotoxic T lymphocytes (CTLs), suggesting that the delivery of OXA and cGAMP via nano-Al(OH)_3_ is a promising strategy. Upon recognition and uptake of antigens by DCs, these cells migrate to tumor-draining lymph nodes to deliver the necessary signals for T cell activation. To further elucidate the effect of nano-Al(OH)_3_-mediated tumor antigen capture on this process, we conducted observations of DCs and T lymphocytes within tumor-draining lymph nodes. Remarkably, in comparison to GO@FA-lip, treatment with AGO@FA-lip resulted in a greater accumulation of MHCⅡ^+^ DCs and CD3^+^CD8^+^ T cells in the paracortex (Fig. 6G). The adsorption and storage of tumor antigens by nano-Al(OH)_3_ create favorable conditions for DCs to recognize and present these antigens [17, 18]. Furthermore, the accumulation of dsDNA in ID8 cells, along with exogenous cGAMP, synergistically activates the cGAS-STING inflammatory cascade, positively influencing the maturation of DCs and the infiltration of T lymphocytes within tumors [58, 59]. In comparison to other treatment groups, there was a notable increase in the infiltration of CD3^+^CD44^+^CD62L^−^ effector memory T (Tem) cells and CD3^+^CD44^+^CD62L^+^ central memory T (Tcm) cells in the paracortical area of lymph nodes in mice treated with AGO@FA-lips (Figure S11B, C). The positivity rate of Tcm in the AGO@FA-lips group was 1.90, 1.63, and 1.88 times higher than that observed in the GO@FA-lips, AO@FA-lips, and AGO@lips groups, respectively (Figure S11D). These collective findings initially indicate that AGO@FA-lip holds promise for reconfiguring the tumor immunosuppressive microenvironment and inducing a robust and enduring antitumor adaptive immune response. This capability is pivotal in effectively preventing postoperative tumor recurrence and metastasis, which is essential for inactivating tumors to achieve an in situ vaccine effect.

### Transcriptomic analysis

**Fig. 7:**
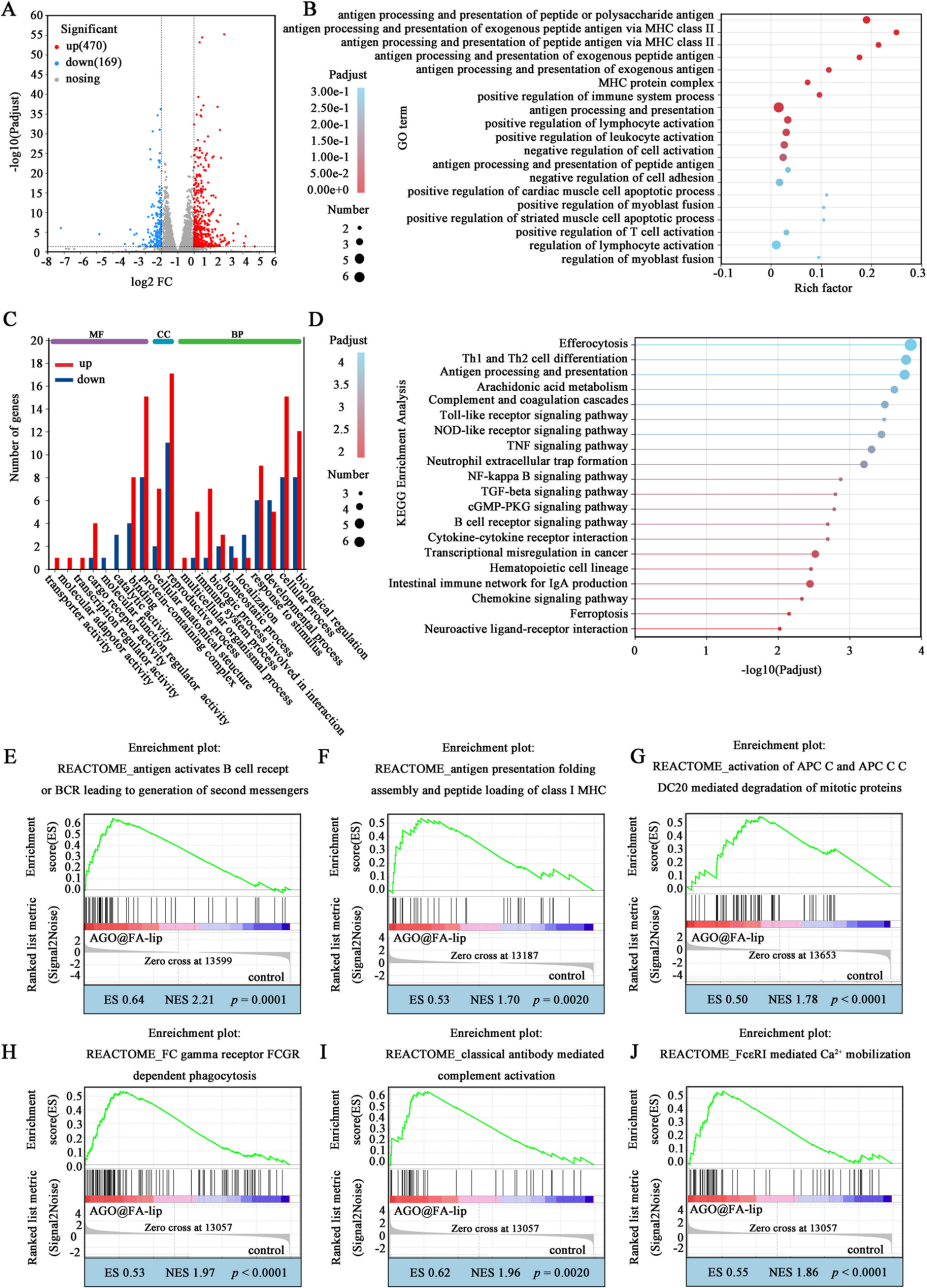
Transcriptomic analysis. **A**. Volcano plot depicting differentially expressed genes (DEGs) in tumor tissues following AGO@FA-lip treatment. **B**, **C**. Gene Ontology (GO) enrichment analysis of DEGs, categorizing functional annotations. **D**. Kyoto Encyclopedia of Genes and Genomes (KEGG) pathway enrich analysis of DEGs, identifying key signaling pathways. E-J. Gene Set Enrichment Analysis (GSEA)

To further elucidate the potential molecular mechanisms by which AGO@FA-lip inhibits tumor growth and induces pathophysiological changes within the TME, we conducted RNA sequencing (RNA-seq) analysis on tumor tissues from ID8 ovarian cancer mouse models. The comprehensive analysis of gene and transcript expression levels provided a quantitative basis for subsequent differential expression analysis (Figure S11E). Among the differentially expressed genes (DEGs) identified between the AGO@FA-lip treatment group and the control group, 470 genes were significantly upregulated, while 169 genes were significantly downregulated (Fig. 7A). Gene Ontology (GO) enrichment analysis revealed that these DEGs are closely associated with various biological processes, including antigen processing and presentation, immune response, and cytokine activity (Fig. 7B). Notably, within the GO biological process (BP) category, genes related to immune system progression were significantly upregulated (Figure S11F). The prevalence of terms associated with stimulation and inflammation underscores the significance of AGO@FA-lip in eliciting immune responses. Furthermore, Gene Ontology (GO) terms pertaining to molecular function (MF), such as the regulation of molecular function activation, transport activation regulation, and transporter activation, corroborate the enhanced cellular activities within the tumor subsequent to AGO@FA-lip administration (Fig. 7C). These findings indicate that AGO@FA-lip treatment has the potential to modulate antigen presentation, immune responses, and inflammatory cytokine levels within the TME, thereby offering valuable insights into the molecular mechanisms through which AGO@FA-lip impedes tumor progression.

To elucidate the principal pathways through which AGO@FA-lip modulates the TME, we conducted a Kyoto Encyclopedia of Genes and Genomes (KEGG) pathway analysis on DEGs. The enrichment analysis revealed activation of pathways associated with apoptosis, T cell differentiation, antigen processing and presentation, Toll-like receptor signaling, and TNF signaling within tumor tissues. These signaling pathways are intimately linked to antigen presentation and specific anti-tumor immune responses (Fig. 7D). Given that AGO@FA-lip effectively activated the cGAS-STING pathway both in vitro and in vivo—a critical process for the activation of innate immunity, we performed gene set enrichment analysis (GSEA) to evaluate whether AGO@FA-lip treatment results in the upregulation or downregulation of tumor-related terms and pathways. The findings indicated that AGO@FA-lip treatment significantly enhanced pathways related to APC recognition and antigen delivery (Fig. 7E-G).

These pathways are essential for eliciting anti-tumor immune responses when utilizing in situ tumor vaccines. Furthermore, treatment with AGO@FA-lip significantly augmented the interferon-mediated inflammatory response within tumor tissues. This was demonstrated by the upregulation of inflammation-associated pathways, such as Fc gamma receptor (FCGR)-dependent phagocytosis, classical antibody-mediated complement activation, and FcεRI-mediated Ca^2+^ mobilization, as identified through GSEA (Fig. 7H-J). The coordinated activation of cytokine signaling pathways underscores a promising strategy for enhancing both innate and adaptive immunity.

The RNA-seq results provide compelling evidence that AGO@FA-lip induces ICD and, following the capture of tumor antigens, further facilitates antigen recognition and presentation. This process enhances the function of CTLs while mitigating the immunosuppressive microenvironment. This dataset significantly advances our understanding of the mechanisms underlying the therapeutic effects of AGO@FA-lip.

### Abscopal effect and vaccine effects

**Fig. 8:**
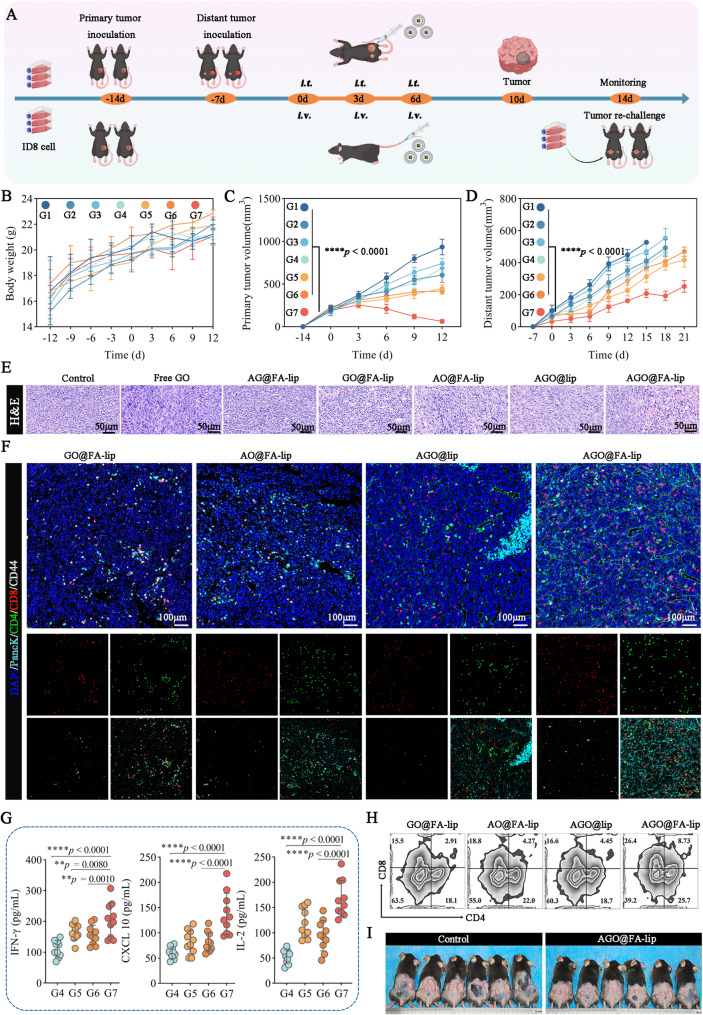
In vivo Abscopal Effect and In Situ Tumor Vaccination Effect. (**A**) Schematic diagram of treatment for distant tumors and tumor re-challenge therapy in vivo. (**B**) Body weight of distant tumors model mice (*n* = 5). (**C**) Volume changes of the primary tumors after treatment (*n* = 5). (**D**) Volume changes of the distant tumors after treatment (*n* = 5). (**E**) H&E staining of the distant tumors. Scale bar 50 μm. (**F**) mIHC of CD4^+^ T cells, CD8^+^ T cells and CD44^+^ T cells of distant tumors. Scale bar 100 μm. (**G**) IFN-γ, CXCL10, and IL-2 cytokine levels in distant tumors (*n* = 5). (**H**) FCM detection of CD3^+^CD4^+^ T cells and CD3^+^CD8^+^ T cells in spleen of distant tumor model mice. (**I**) Distant tumor mice on the 10th day of treatment. * *p* < 0.05, **p < 0.01, ***p < 0.001, ****p < 0.0001

The vaccine effect of tumors inactivated by AGO@FA-lip was evaluated in bilateral subcutaneous tumor transplantation models and tumor re-challenge models. The detailed procedures for processing and evaluation are depicted in Fig. 8A. To more accurately assess the systemic anti-tumor effects of AGO@FA-lip on primary tumors, intratumoral injections were administered to mice, specifically targeting primary tumors to eliminate the influence of OXA and cGAMP on distant tumor growth. Throughout the treatment period, no significant drop in body weight was observed in tumor-bearing mice across all groups (Fig. 8B). By day 12, the average volume of primary tumors in the AGO@FA-lip group was notably smaller compared to other groups (*p* < 0.0001, Fig. 8C). Mice in the control, Free GO, AG@FA-lip, and GO@FA-lip groups reached the experimental endpoint sooner due to accelerated tumor growth. By day 21, the average volume of *distant tumors* in the AGO@FA-lip group remained significantly smaller than those in the AO@FA-lip and AGO@lip groups (*p* < 0.0001, Fig. 8D). More significantly, AGO@FA-lip extended the duration required for distant tumors to achieve a predetermined endpoint volume of 500 mm³ (*p* < 0.0001, Figure S12A). H&E staining revealed an increase in infiltrating inflammatory cells and necrotic regions within the distant tumor tissues in the AGO@lip group (Fig. 8E). These findings suggest that the treatment of primary tumors with AGO@FA-lip effectively activated systemic anti-tumor responses and inhibited the growth of distant tumors. To investigate the antitumor efficacy of tumor tissues inactivated by AGO@FA-lip as a tumor vaccine, an experimental tumor recurrence model was developed using tumor-bearing mice. Mice treated with AGO@FA-lip demonstrated resistance to tumor re-challenge, exhibiting antitumor rates of 100% and 87.50% at 20 and 30 days, respectively. Notably, only 12.5% of the mice developed new subcutaneous tumors within 30 days following re-inoculation with ID8 cells (Figure S12B).

 The primary objective of cancer vaccination is to stimulate or augment activated T cell responses targeting tumor-associated antigens [60, 61]. To further elucidate the effects of AGO@FA-lip-mediated inactivation of primary tumors on subsequent anti-tumor immunity, we assessed alterations in the immune microenvironment within distant tumors. Quantitative cell analysis revealed a significant elevation in CD3^+^ T cells (*p* = 0.0020), CD4^+^ T cells (*p* = 0.0010), CD8^+^ T cells (*p* < 0.0001), and CD44^+^ T cells (*p* = 0.0010) compared to the control group, whereas FoxP3^+^ Treg cells exhibited a decrease (*p* = 0.0100) (Figure S12C). mIHC results revealed that distant tumors in the AGO@FA-lip group exhibited the highest numbers of CD3^+^CD4^+^ T cells, CD3^+^CD8^+^ T cells, and CD3^+^CD44^+^ T cells (Fig. 8F). The upregulation of CD44 expression facilitates the interaction between T cells and DCs, enhances the transmission of activation signals from T cells, and promotes T cell proliferation and function [62]. Furthermore, CD44 is crucial in forming and maintaining long-term immune memory, enabling T cells to rapidly initiate a response upon re-encountering the same antigen [63]. Concurrently, increased levels of IFN-γ, CXCL10, and IL-2 were detected in the distant tumors of the AGO@FA-lip group (Figs. 8G, S13A), suggesting an enhancement of specific anti-tumor immune responses within these distant tumors. Additionally, an increase in CD3^+^CD4^+^ T cells, and CD3^+^CD8^+^ T cells was observed in the spleens of mice in the AGO@FA-lip group, indicating that the inactivation of primary tumors activated a systemic anti-tumor immune response in these mice (Fig. 8H, S13B). Compared with untreated mice, the volumes of both primary and distant tumors in mice of the AGO@FA-lip group were significantly reduced on the tenth day of treatment (Fig. 8I). These findings establish a laboratory foundation for the use of AGO@FA-lip in tumor inactivation and the development of in situ vaccines.

## Conclusion

In this study, AGO@FA-lip exhibited specificity in targeting tumor cells via folic acid molecules, thereby inducing ICD and facilitating the exposure of tumor antigens and the release of dsDNA. The inactivation of the tumor and the acquisition of tumor antigens represent the preliminary steps in the development of an in vivo vaccine. The subsequent capture of tumor antigens by nano-Al(OH)_3_ enhanced the recognition and presentation of these antigens by DCs, leading to a more effective activation of T lymphocytes. This capture of tumor antigens constitutes the second step in constructing an in situ vaccine. The combined administration of exogenous cGAMP and dsDNA synergistically amplified STING signaling pathways, resulting in the remodeling of the tumor inflammatory microenvironment. This remodeling is a crucial step in ensuring the efficacy of tumor vaccines. In a murine model of ovarian cancer, AGO@FA-lip modulated critical biological processes, effectively inactivating primary tumors while simultaneously activating systemic-specific anti-tumor immunity, thereby suppressing both metastasis and recurrence.

AGO@FA-lip employed its complementary mechanisms to enhance therapeutic efficacy. In contrast to the mere co-delivery of STING agonists and DNA-damaging agents, the nano-Al(OH)_3_-mediated antigen capture significantly augmented AGO@FA-lip’s capacity to induce DC maturation, activate T cells, and facilitate antigen presentation by DCs to T lymphocytes within tumor-draining lymph nodes. Transcriptomic analysis further highlighted the processes of antigen processing and presentation, T cell differentiation, and the activation of the TNF inflammatory signaling pathway. Although the adjuvant effect of aluminum hydroxide is well-documented, its underlying mechanism remains elusive. The activation of innate immune pathways by aluminum-based adjuvants may represent an auxiliary mechanism through which AGO@FA-lip promotes anti-tumor immunity. Recent studies on aluminum-based nanodrug delivery systems have underscored the excellent biological safety profile of aluminum [64–67]. Unlike these aluminum-based drug-loaded systems, we have enhanced the tumor-targeting capability of AGO@FA-lip through molecular targeting technology, thereby reducing the non-specific distribution of aluminum and platinum during intravenous administration. The aluminum content of AGO@FA-lip (9.53 µg/mL) is below the safety threshold established for aluminum adjuvants by the World Health Organization and the European Union (1.25 mg/dose), China (0.35–3.00 mg/mL), and the United States (0.85 mg/dose) [68–70]. All constituent materials of AGO@FA-lip have received FDA approval, enhancing its potential for clinical translation compared to many other newly developed biomaterials.

In essence, AGO@FA-lip effectively acquires tumor antigens while simultaneously inactivating tumors, which constitutes the core mechanism of the vaccine. Concurrently, AGO@FA-lip enhances the innate immune STING signaling pathway by delivering cGAMP, thereby remodeling the tumor immune microenvironment and mitigating barriers to subsequent anti-tumor immune activation. Unlike the in situ vaccine construction strategies that primarily focus on inducing ICD, the antigen capture and STING pathway activation by AGO@FA-lip exert a more favorable influence on antigen recognition by DCs, T cell activation, and the remodeling of the immune microenvironment. This study presents a rational design of a nanosystem aimed at developing in situ tumor vaccines and enhancing vaccine efficacy. The AGO@FA-lip formulation shows potential for personalized treatment of metastatic and recurrent tumors.

AGO@FA-lip exhibits the capability to actively target tumors with high expression of folate receptors; however, its antitumor efficacy may be restricted in tumors with low folate receptor expression. The absence of specific tumor antigen recognition by nano-Al(OH)_3_ leads to inevitable non-specific protein adsorption, including serum proteins, during in vivo applications. Although AGO@FA-lip has shown promising antitumor effects and the ability to remodel the immune microenvironment, the impact of non-tumor protein adsorption on AGO@FA-lip-mediated antitumor immunity remains uncertain. Beyond effectively inactivating tumors and exposing antigens, optimal antigen-trapping nanoprobes should possess the capability to specifically recognize and capture tumor antigens. While the concurrent use of immune checkpoint inhibitors (ICIs) is currently advocated in tumor immunotherapy, the effectiveness of combining in situ tumor vaccines developed with AGO@FA-lip and ICIs has yet to be determined. Furthermore, the molecular mechanisms governing the interactions among captured antigens, nano-Al(OH)_3_, and APCs are not yet fully elucidated. The molecular weight and type of antigens significantly influence the uptake pathways of APCs. Our future research will concentrate on optimizing targeting strategies, enhancing the specificity of antigen capture, improving the combined efficacy with ICIs, and advancing the transport of captured antigens within the lymphatic system, as well as their subsequent presentation to T lymphocytes.

## Supplementary Information

Supplementary Material 1

Supplementary Material 2

## Data Availability

No datasets were generated or analysed during the current study.

## Abbreviations

OXA: Oxaliplatin
TSA: Tumor-specific antigens
TAA: Tumor-associated antigens
TME: Tumor microenvironment
ICD: Immunogenic cell death
dsDNA: Double-stranded DNA
MHC: Major histocompatibility complex
APC: Antigen-presenting cells
HGSC: High-grade serous carcinoma
IFN-I: Interferon type 1
STING: Stimulator of the interferon genes
BMDC: Bone marrow-derived dendritic cell
mIHC: Multiplex immunofluorescence staining
CTLs: Cytotoxic T lymphocytes
KEGG: Kyoto encyclopedia of genes and genomes
GO: Gene ontology
GSEA: Gene set enrichment analysis
