# Supplementary material for: Folic acid-modified antigen-trapping nanoprobes for developing in situ tumor vaccines to inhibit metastasis and recurrence of ovarian cancer
Source: J Nanobiotechnology. 2026 May 11;24:620. doi: 10.1186/s12951-026-04537-5 (PMC13335270; doi:10.1186/s12951-026-04537-5)

**Supporting Information 2**

**Folic Acid-Modified Antigen-Trapping Nanoprobes for Developing In Situ Tumor Vaccines to Inhibit Metastasis and Recurrence of Ovarian Cancer**

Xiaowen Zhong^1,2,3*^, Tao Pu^1,2^, Ying Cheng^1,2^, Yan Li^1^, Qi Wang^3^, Bin Wang ^1*^


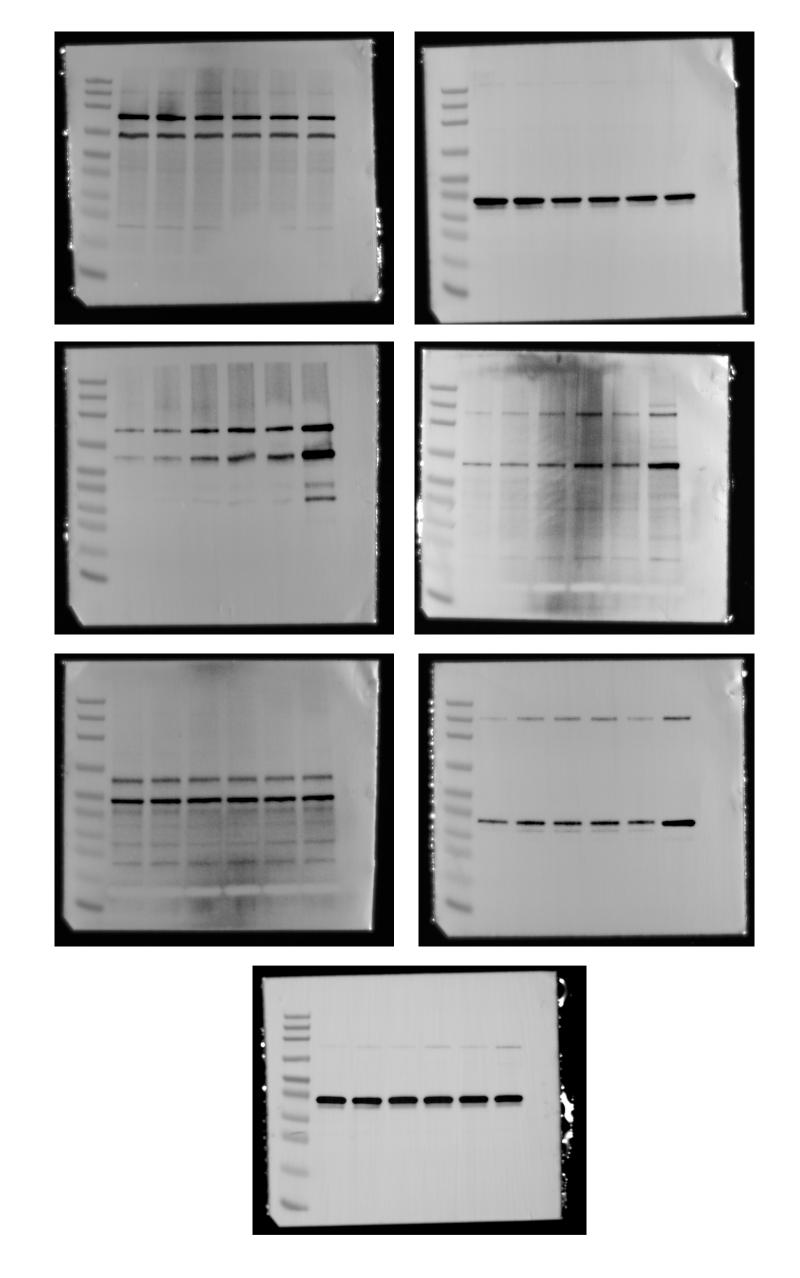

Supplement: Supplementary file 1 — Supplementary Material 1 [file 12951_2026_4537_MOESM1_ESM.docx]
