# Supplementary material for: Folic acid-modified antigen-trapping nanoprobes for developing in situ tumor vaccines to inhibit metastasis and recurrence of ovarian cancer
Source: J Nanobiotechnology. 2026 May 11;24:620. doi: 10.1186/s12951-026-04537-5 (PMC13335270; doi:10.1186/s12951-026-04537-5)
Supplement: Supplementary file 2 — Supplementary Material 2 [file 12951_2026_4537_MOESM2_ESM.docx]

**Supporting Information 1**

**Folic Acid-Modified Antigen-Trapping Nanoprobes for Developing In Situ Tumor Vaccines to Inhibit Metastasis and Recurrence of Ovarian Cancer**

Xiaowen Zhong^1,2,3*^, Tao Pu^1,2^, Ying Cheng^1,2^, Yan Li^1^, Qi Wang^3^, Bin Wang ^1*^

Table S1. Materials, Antibodies and Chemicals Information

| Materials, Antibodies and Chemicals (Cat.) | SOURCE |
| --- | --- |
| DPPC, DSPE-PEG2000-FA | Ruixi Biotechnology  (Xian, China) |
| Cholesterol | Sigma Aldrich |
| Oxaliplatin (OXA) ([HY-17371)](https://www.medchemexpress.cn/Oxaliplatin.html) | MedChemExpress |
| cGAMP | MedChemExpress |
| Nano aluminum hydroxide | Ruixi Biotechnology |
| Pacific Blue^TM^ anti-mouse CD45 Antibody (#157212) | BioLegend |
| PE anti-mouse CD3 Antibody (#100206) | BioLegend |
| FITC anti-mouse CD3 Antibody (#100203) | BioLegend |
| PC5.5 anti-mouse CD4 Antibody (#100206) | BioLegend |
| APC anti-mouse CD8a Antibody (#162306) | BioLegend |
| FITC anti-mouse CD44 Antibody (#156008) | BioLegend |
| APC-Cy7 anti-mouse CD62L antibody (#104428) | BioLegend |
| PE anti-mouse CD11c Antibody (#117308) | BioLegend |
| FITC anti-mouse CD80 Antibody (#104705) | BioLegend |
| PC5.5 anti-mouse CD86 Antibody (#159211) | BioLegend |
| APC anti-mouse I-A/I-E Antibody (#107614) | BioLegend |
| APC anti-mouse H-2kb/ SIINFEKL Antibody (#141606) | BioLegend |
| APC anti-mouse CD25 Antibody (#113708) | BioLegend |
| PE anti-mouse CD69 Antibody(#104507) | BioLegend |
| Zombie NIR™ Fixable Viability Kit (#423106) | BioLegend |
| STING rabbit antibodies (#13647) | Cell Signaling Technology |
| P-STING rabbit antibodies (#50907) | Cell Signaling Technology |
| TBK1 rabbit antibodies (#82382) | Cell Signaling Technology |
| P-TBK1rabbit antibodies (#5483) | Cell Signaling Technology |
| IRF3 rabbit antibodies (AF2485) | Affinity Biosciences |
| P-IRF3 rabbit antibodies (AF1594) | Affinity Biosciences |
| GADPH Antibody | Servicebio |
| HRP-conjugated Goat Anti-Rabbit IgG (H+L) | Servicebio |
| ATP Chemiluminescence Assay Kit (E-BC-F002) | Elabscience |
| [Mouse HMGB-1 ELISA Kit](https://www.elabscience.cn/p-mouse_hmgb_1_highmobility_group_protein_b1_elisa_kit-e_el_m0676) (E-EL-M0676) | Elabscience |
| [Mouse HSP-70/HSPA9ELISA Kit](https://www.elabscience.cn/p-mouse_hsp_70_hspa9_heat_shock_protein_70_elisa_kit-e_el_m0619) (E-EL-M0619) | Elabscience |
| [MS Mouse IL-6 ELISA Kit](https://www.elabscience.cn/p-ms_mouse_il_6_interleukin_6_elisa_kit-350469.html) (E-MSEL-M0001) | Elabscience |
| [Mouse IFN-β ELISA Kit](https://www.elabscience.cn/p-mouse_ifn_interferon_beta_elisa_kit-e_el_m0033) (E-EL-M0033) | Elabscience |
| [Mouse IP-10/CXCL10 ELISA Kit](https://www.elabscience.cn/p-mouse_ip_10_cxcl10_interferon_gamma_induced_protein_10kda_elisa_kit-e_el_m0021) (E-EL-M0021) | Elabscience |
| Mouse IL-12 p70 ELISA Kit (433607) | BioLegend |
| [MS Mouse IL-12 ELISA Kit](https://www.elabscience.cn/p-ms_mouse_il_12_interleukin_12_elisa_kit-350472.html) (E-MSEL-M0004) | Elabscience |
| [Mini Sample Mouse IFN-γ ELISA Kit](https://www.elabscience.cn/p-mini_sample_mouse_ifn_interferon_gamma_elisa_kit-e_msel_m0007) (E-MSEL-M0007) | Elabscience |
| [MS Mouse TNF-α](https://www.elabscience.cn/p-ms_mouse_tnf_alpha_tumor_necrosis_factor_alpha_elisa_kit-350470.html) [ELISA Kit (](https://www.elabscience.cn/p-ms_mouse_tnf_alpha_tumor_necrosis_factor_alpha_elisa_kit-350470.html)E-MSEL-M0002) | Elabscience |
| [Mini Sample Mouse IL-10 ELISA Kit](https://www.elabscience.cn/p-mini_sample_mouse_il_10_interleukin_10_elisa_kit-e_msel_m0031) (E-MSEL-M0031) | Elabscience |
| [Mouse TGF-β2 ELISA Kit](https://www.elabscience.cn/p-mouse_tgf_2_transforming_growth_factor_beta_2_elisa_kit-e_el_m1191) (E-EL-M1191) | Elabscience |
| [Mini Sample Mouse VEGF-A ELISA Kit](https://www.elabscience.cn/p-mini_sample_mouse_vegf_a_vascular_endothelial_cell_growth_factor_a_elisa_kit-e_msel_m0005) (E-MSEL-M0005) | Elabscience |
| CFSE Cell Division Tracker Kit (423801) | BioLegend |
| [PCNA Monoclonal Antibody (](https://www.elabscience.cn/p-pcna_monoclonal_antibody-77926.html)E-AB-22001) | Elabscience |
| [TUNEL Assay Kit (HRP-DAB) (](https://www.elabscience.cn/p-tunel_assay_kit_hrp_dab_-216233.html) E-CK-A331) | Elabscience |
| [Anti-Calreticulin antibody (ab92516)](https://www.abcam.cn/calreticulin-antibody-epr3924-er-marker-ab92516.html) | Abcam |
| [Anti-HMGB1 antibody (ab79823)](https://www.abcam.cn/hmgb1-antibody-epr3507-ab79823.html) | Abcam |
| CK-Pan (ab7753) | Abcam |
| HSP70 (AFRM0031) | Aifang Biotechnolog |
| [Anti-CD3 antibody](https://www.abcam.cn/cd3-antibody-sp162-ab135372.html) (AFRM0030) | Aifang Biotechnology |
| [Anti-CD4 antibody](https://www.abcam.cn/cd4-antibody-cal4-ab237722.html) (AFRM0003) | Aifang Biotechnology |
| [Anti-CD8 antibody](https://www.abcam.cn/cd8-alpha-antibody-epr21769-ab217344.html) (AFRM0004) | Aifang Biotechnology |
| Anti-CD44 antibody (AFRM0172) | Aifang Biotechnology |
| Anti-CD20 antibody (AFRM0016) | Aifang Biotechnology |
| Anti-F4/80 antibody (AFRM0005) | Aifang Biotechnology |
| FoxP3 (AFRM0359) | Aifang Biotechnology |
| Ki67 (AFRP0030) | Aifang Biotechnology |
| Polymer-HRP anti-mouse/rabbit universal secondary antibody IgG (AFIHC001) | Aifang Biotechnology |
| dsDNA maker antibody (sc-58749) | Santa Cruz Biotechnology |
| [Rabbit Anti-Mouse IgG H&L (HRP) (ab6728)](https://www.abcam.cn/rabbit-mouse-igg-hl-hrp-ab6728.html) | Abcam |
| Goat Anti-Rabbit IgG H&L (Alexa Fluor® 488) preadsorbed (ab150081) | Abcam |
| [Goat Anti-Rabbit IgG H&L (Cy5 ®) preadsorbed (ab6564)](https://www.abcam.cn/goat-rabbit-igg-hl-cy5--preadsorbed-ab6564.html) | Abcam |

| [Bradford Assay Kit (ab102535)](https://www.abcam.cn/bradford-assay-kit-ab102535.html) | Abcam |
| --- | --- |
| Bovine serum albumin (BSA)( A8010) | Solarbio |
| OVA-FITC (SF069) | Solarbio |
| Mouse Tumor Infiltrating Lymphocyte Separation Solution Kit | Solarbio |
| Fetal bovine serum (10099141) | Gibco |
| DMEM/HIGH | Hyclone |
| RPMI1640 | Hyclone |
| PBS .0067M, 500ML | Hyclone |
| 4% paraformaldehyde fixativeSolution (P0099) | Beyotime Biotechnology |
| Antifade Mounting Medium (P0126) | Beyotime Biotechnology |
| ATP Assay Kit (S0026B) | Beyotime Biotechnology |
| Cell Counting Kit-8 (CCK8) (C0038) | Beyotime Biotechnology |
| Reactive Oxygen Species Assay Kit (DCFH-DA)(S0033M) | Beyotime Biotechnology |
| LDH Cytotoxicity Assay Kit (C0016) | Beyotime Biotechnology |
| DiI (C1036) | Beyotime Biotechnology |
| DiO (C19935) | Beyotime Biotechnology |
| DAPI (C1005) | Beyotime Biotechnology |
| Hoechst 33342 Live Cell Stain (C1029) | Beyotime Biotechnology |
| Calcein-AMand propidium iodide (sc-203865, sc-3541) | Santa Cruz Biotechnology |
| [Annexin V-FITC/PI Apoptosis Kit](https://www.elabscience.cn/p-annexin_v_fitc_pi_apoptosis_kit-203045.html) (E-CK-A211) | Elabscience |
| Triton-x100 (X100) | Sigma Aldrich |
| Fluoromount (F4680) | Sigma Aldrich |
| ACK Lysis Buffer | Solarbio |
| Fibrinogen | Solarbio |

Table S2. Physicochemical properties of the prepared nanoprobes

| NPs | Size (nm) | Zeta Potential (mV) | PDI |
| --- | --- | --- | --- |
| nano Al(OH)_3_ | 95.43±2.32 | 41.38±1.24 | 0.13±0.02 |
| GO@FA-lip | 168.31±5.29 | -29.73±2.05 | 0.11±0.04 |
| AG@ FA-lip | 212.77±4.74 | -20.46±1.59 | 0.12±0.03 |
| AO@ FA-lip | 200.57±7.42 | -25.66±1.99 | 0.09±0.01 |
| AGO@ lip | 223.13±6.14 | -24.42±1.90 | 0.10±0.03 |
| AGO@ FA-lip | 216.19±5.34 | -27.51±2.14 | 0.11±0.02 |

Table S3. Drug encapsulation efficiency (EE) and loading efficiency (LE) of different nanoprobes

| NPs | EE%  of  cGAMP | LE%  of  cGAMP | EE%  of  OXA | LE%  of  OXA | EE%  of  Al(OH)_3_ | LE%  of  Al(OH)_3_ |
| --- | --- | --- | --- | --- | --- | --- |
| GO@FA-lip | 46.09±6.26 | 2.37±0.41 | 71.28±2.41 | 8.93±0.78 | - | - |
| AG@ FA-lip | 75.05±3.37 | 4.06±0.25 | - | - | 53.1±2.70 | 14.35±0.51 |
| AO@ FA-lip | - | - | 62.33±1.73 | 8.42±0.23 | 48.62±3.16 | 13.14±0.68 |
| AGO@ lip | 70.81±4.11 | 3.83±0.36 | 60.17±2.04 | 8.13±0.59 | 55.07±4.72 | 14.88±0.74 |
| AGO@ FA-lip | 68.42±5.17 | 3.56±0.22 | 58.9±2.19 | 7.96±0.46 | 52.41±5.02 | 14.16±0.80 |


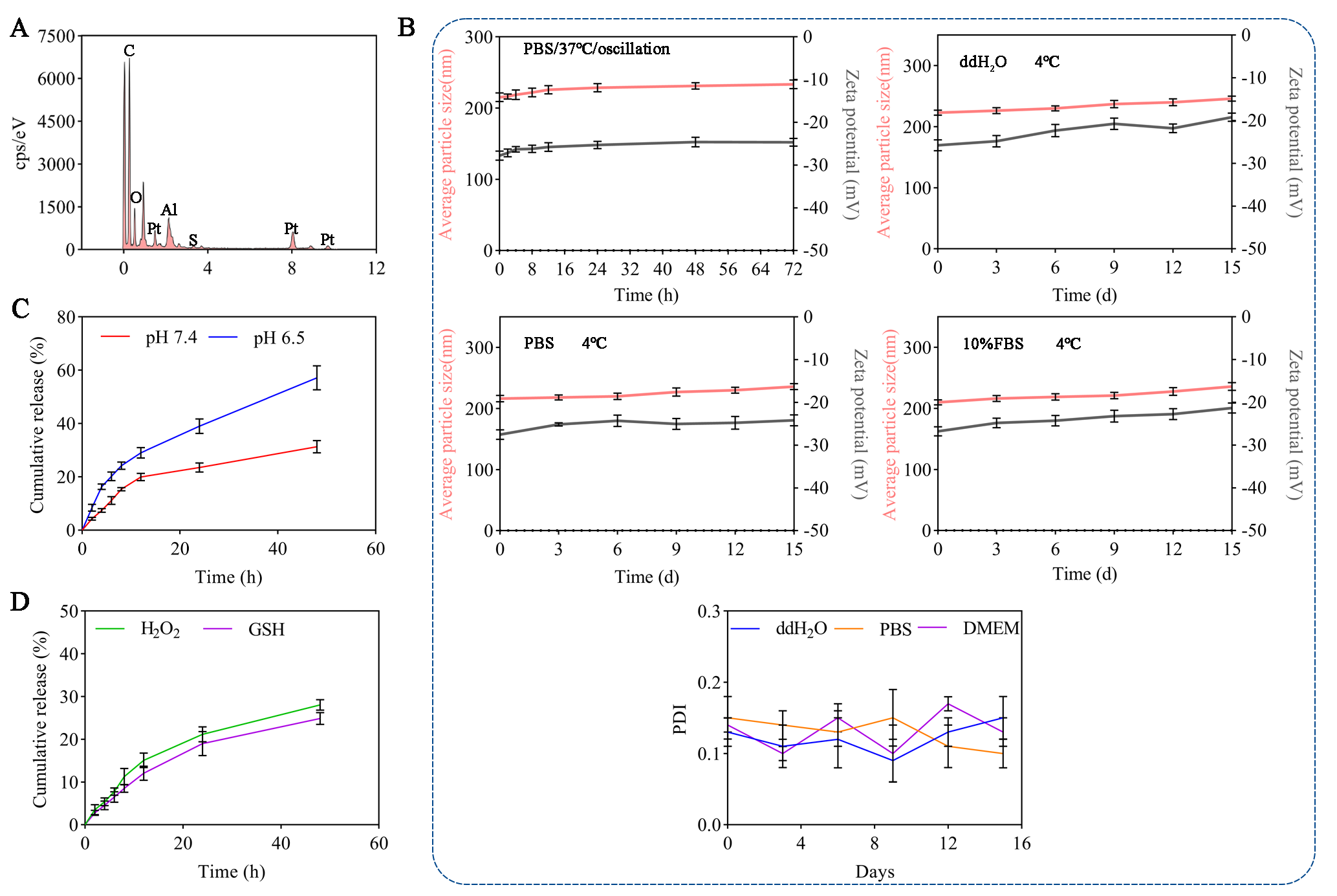


Figure S1. A. Map Sum Spectrum of AGO@FA-lip. B. Changes in particle size, zeta potential and PDI of AGO@FA-lip in ddH_2_O, PBS, and DMEM over 15 days (n = 3). C. Cumulative release rate of OXA from AGO@ FA-lip at pH 6.5 and pH 7.4 (n = 3). D. Cumulative release rate of OXA from AGO@ FA-lip at H_2_O_2_ and GSH (n = 3). * *p* < 0.05 , ***p* < 0.01, ****p* < 0.001, *****p* < 0.0001.


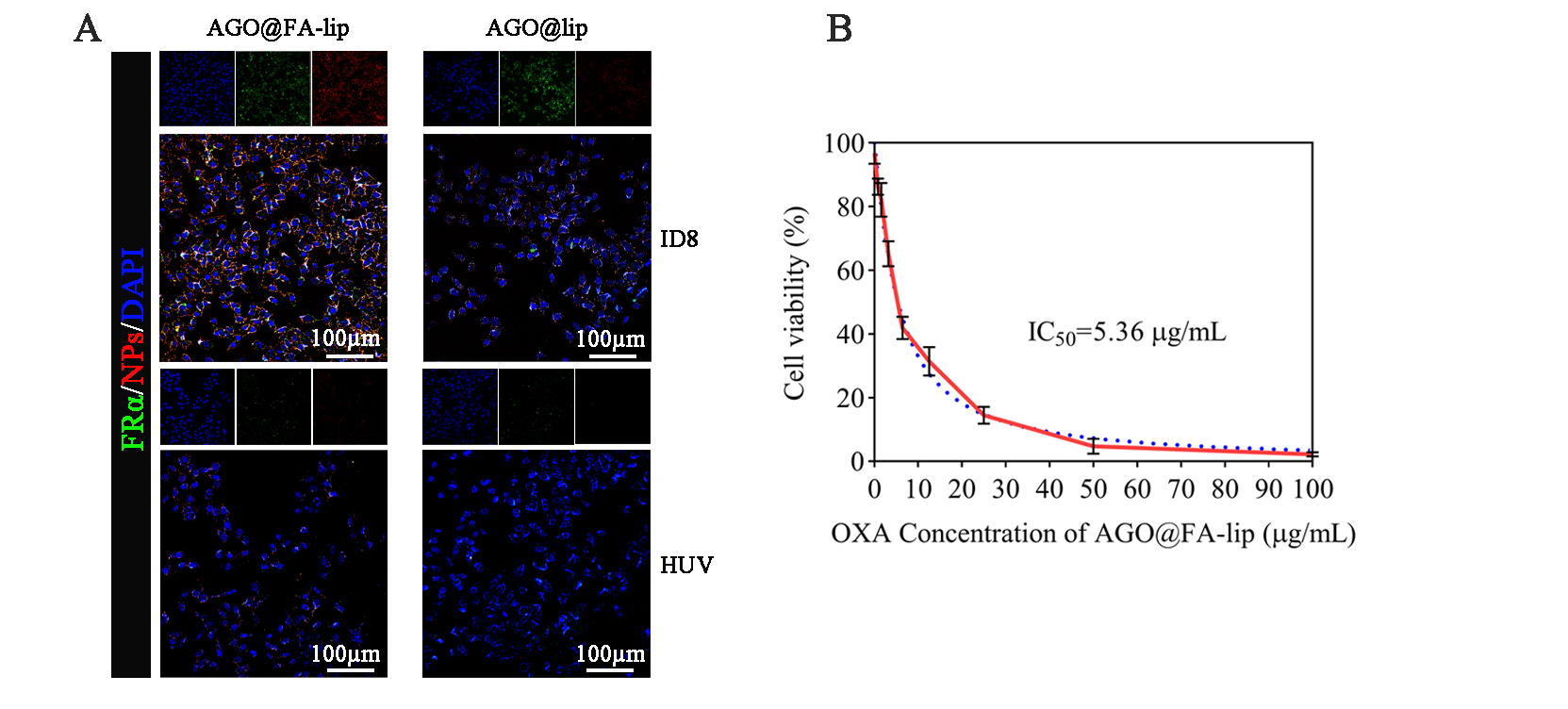


Figure S2. A. CLSM were used to evaluate the uptake of AGO@ FA-lip or AGO@lip by ID8 cells and HUV cells. Scale bar 100 µm. B. Cell viability and IC_50_ of ID8 cells after incubation with AGO@ FA-lip containing different concentrations of OXA(n = 3). * *p* < 0.05 , ***p* < 0.01, ****p* < 0.001, *****p* < 0.0001.


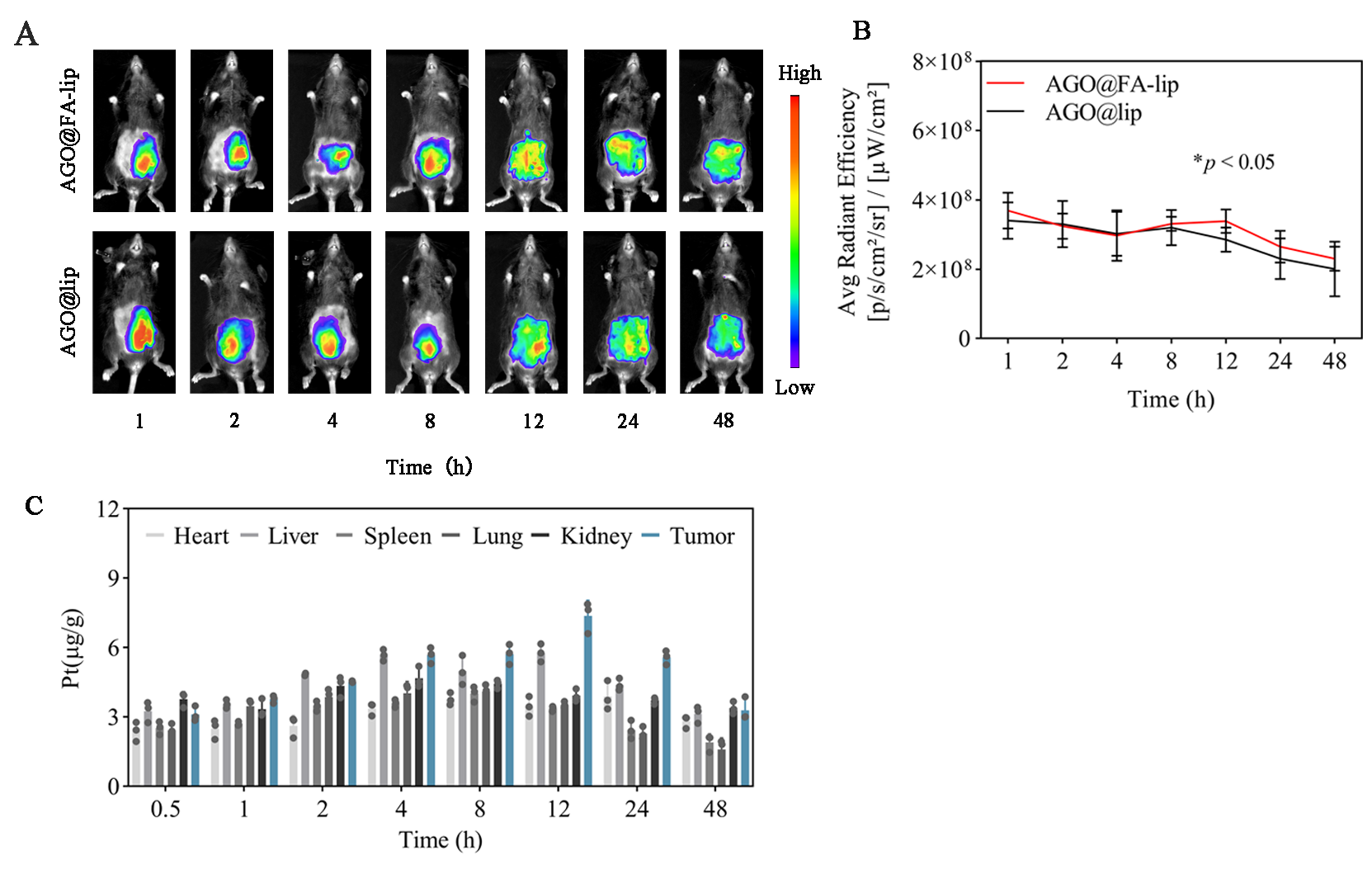


Figure S3. A. *In vivo* fluorescence imaging and statistical analysis of average fluorescence intensity in intraperitoneal metastasis tumor mice within 48 hours post-intraperitoneal administration of AGO@FA-lip or AGO@lip (n = 3). B. Tumor fluorescence imaging of tumor-bearing mice at different time points following intraperitoneal administration (n = 3). C. ICP-MS analysis of OXA (Pt) concentration in major organs and tumor tissues of mice within 48 hours after intravenous injection of AGO@FA-lip (n = 3). * *p* < 0.05 , ***p* < 0.01, ****p* < 0.001, *****p* < 0.0001.


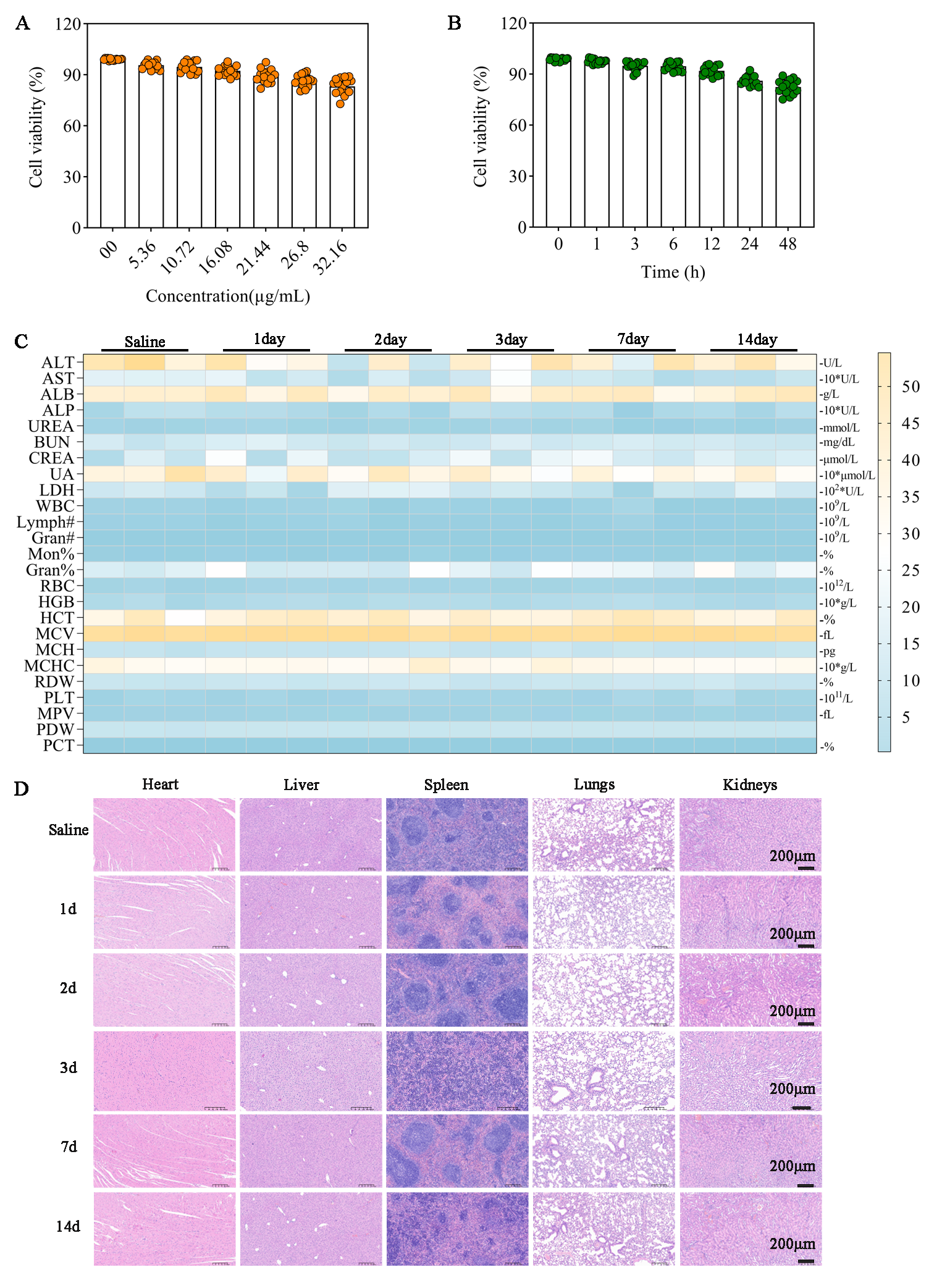


Figure S4. A.The cytotoxicity of AGO@FA-lips at varying concentrations (0, 5.36, 10.72, 16.08, 21.44, 26.8, 32.16 μg/mL) on HUVECs (n = 10). B. The cytotoxic effects were evaluated following the co-incubation of HUVECs with AGO@FA-lips at the therapeutic concentration over different time (1, 2, 3, 6, 12, 24 hours) (n = 10). C. Blood cell, blood glucose, liver function, renal function, and cardiac function tests of mice at different times after intravenous injection of AGO@FA-lip (n = 3). D. H&E staining of the main organs of mice at different times after intravenous injection of AGO@FA-lip. Scale bar 200 µm. * *p* < 0.05, ***p* < 0.01, ****p* < 0.001, *****p* < 0.0001.


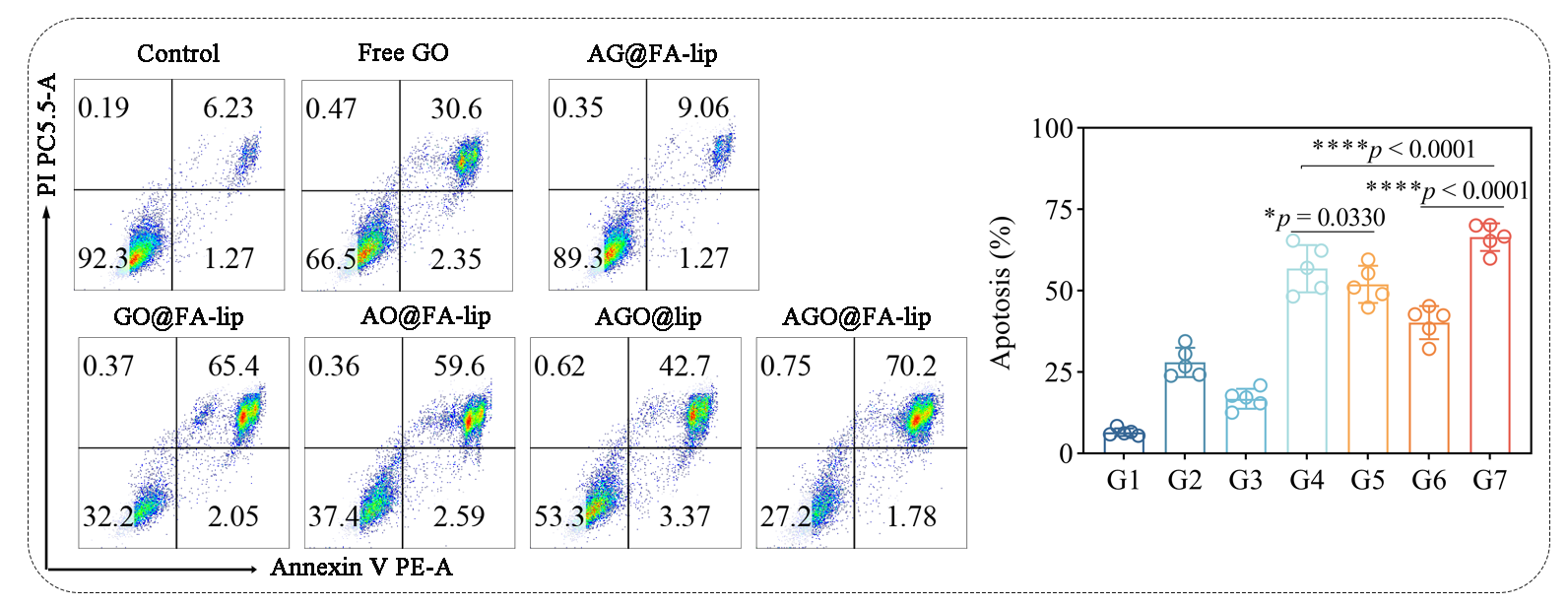


Figure S5. Flow cytometry detection of AGO@FA-lip mediated apoptosis in ID8 cells and statistical analysis (n = 5). * *p* < 0.05 , ***p* < 0.01, ****p* < 0.001, *****p* < 0.0001.


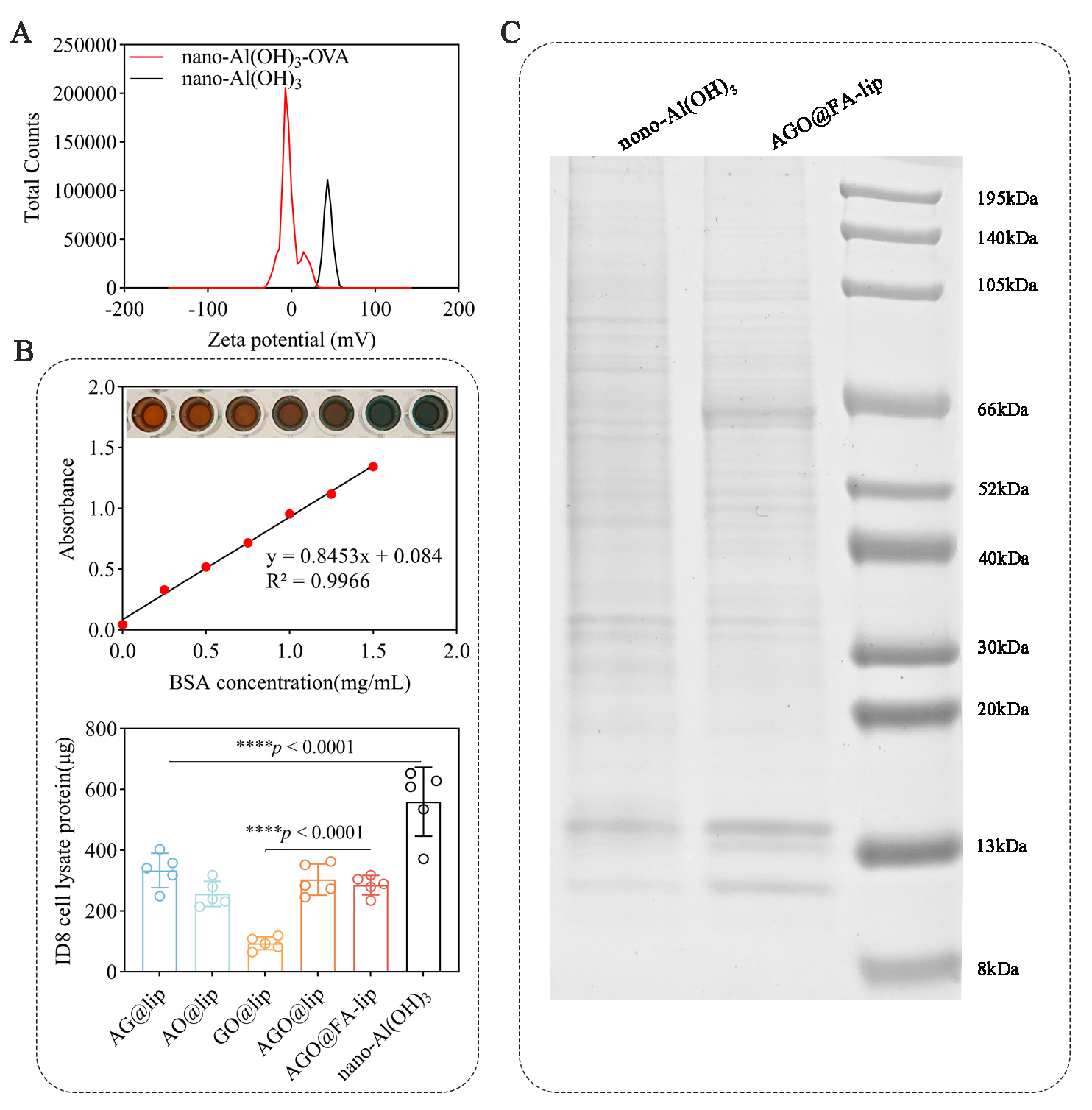


Figure S6. A. Zeta potential changes of nano-Al(OH)_3_ after protein adsorption. B. The capture efficiency of tumor lysate proteins by the nanoprobes was quantified using the Bradford method (n = 5). C. In the serum co-incubation experiment, distinct differences were noted in the protein profiles captured by nano-Al(OH)_3_ and AGO@FA-lip. * *p* < 0.05, ***p* < 0.01, ****p* < 0.001, *****p* < 0.0001.

Table S4. Tumor antigens captured by AGO@FA-lip

| Capture antigens | UniProt ID | Gene | #PSMs | Unique Peptides | Annotated Sequence |
| --- | --- | --- | --- | --- | --- |
| AHNK | [Q09666](http://www.uniprot.org/uniprot/Q09666) | AHNAK | 132 | 85 | GKGGVTGSPEASISGSK |
| ANXA2 | [P07355](http://www.uniprot.org/uniprot/P07355) | ANXA2 | 26 | 9 | LEGDHSTPP |
| BCAP31 | [Q53G72](http://www.uniprot.org/uniprot/Q53G72) | BCAP31 | 8 | 4 | YMEENDQLK |
| BRAF | [P15056](http://www.uniprot.org/uniprot/P15056) | BRAF | 3 | 2 | RDSSDDWEIPDG |
| CASP8 | [Q14790](http://www.uniprot.org/uniprot/Q14790) | CASP8 | 5 | 2 | GRAQISAYRVM |
| CTNNB1 | P35222 | CTNNB1 | 4 | 2 | TmQNTNDVETAR |
| EEF2 | [P13639](http://www.uniprot.org/uniprot/P13639) | EEF2 | 28 | 11 | EGALcEENmR |
| H3F3A | [P84243](http://www.uniprot.org/uniprot/P84243) | H3-3A | 7 | 2 | VTImPK |
| Histone H1.1 | [Q02539](https://www.uniprot.org/uniprotkb/Q02539/entry) | H1-1 | 4 | 2 | DATPEScSLLPQNEER |
| Histone H1.2 | [P16403](https://www.uniprot.org/uniprotkb/P16403/entry) | H1-2 | 8 | 2 | KATGAATPK |
| Histone H1.3 | [P16402](https://www.uniprot.org/uniprotkb/P16402/entry) | H1-3 | 9 | 3 | ASGPPVSELITK |
| Histone H1.4 | [P10412](https://www.uniprot.org/uniprotkb/P10412/entry) | H1-4 | 6 | 2 | KPAAAAGAK |
| Histone H1.5 | [P16401](https://www.uniprot.org/uniprotkb/P16401/entry) | H1-5 | 3 | 3 | KPAGATPK |
| Histone H2A 2-C | [Q64523](https://www.uniprot.org/uniprotkb/Q64523/entry) | H2ac20 | 4 | 3 | SQTAELFAK |
| Histone H2B | [Q93079](https://www.uniprot.org/uniprotkb/Q93079/entry) | H2BC9 | 25 | 4 | AVTKYTSSK |
| Histone H4 | [P62806](https://www.uniprot.org/uniprotkb/P62806/entry) | Hist1h4m | 4 | 2 | VSQALEQQAR |
| HMGB1 | [P09429](https://www.uniprot.org/uniprotkb/P09429/entry) | HMGB1 | 10 | 2 | TYIPPKGETK |
| HMG-I/HMG-Y | [A0A338P6F6](https://www.uniprot.org/uniprotkb/A0A338P6F6/entry) | Hmga1 | 3 | 2 | GIPAPEEERTR |
| HMGI-C | [P52926](https://www.uniprot.org/uniprotkb/P52926/entry) | HMGA2 | 12 | 3 | KQQQEPTcEPSPK |
| HNRPL | [P14866](http://www.uniprot.org/uniprot/P14866) | HNRNPL | 22 | 6 | HYEGRRMGPPVG |
| HSP 90-α | [P07901](https://www.uniprot.org/uniprotkb/P07901/entry) | Hsp90aa1 | 28 | 5 | TTPSYVAFTDTER |
| HSP 90-β | [P08238](https://www.uniprot.org/uniprotkb/P08238/entry) | HSP90AB1 | 38 | 8 | SLVSVTK |
| HSPA1A | [P08107](http://www.uniprot.org/uniprot/P08107) | HSPA1A | 18 | 6 | VEIIANDQGNR |
| HSPA1B | [Q59EJ3](http://www.uniprot.org/uniprot/Q59EJ3) | HSPA1B | 50 | 11 | MVQEAEK |
| HSPB1 | [P04792](http://www.uniprot.org/uniprot/P04792) | HSPB1 | 7 | 5 | QLSSGVSEIR |
| JUP | [P14923](http://www.uniprot.org/uniprot/P14923) | JUP | 4 | 4 | TmQNTSDLDTAR |
| LRP1 | [Q07954](http://www.uniprot.org/uniprot/Q07954) | LRP1 | 5 | 4 | cPTGFTGPK |
| PA2G4 | [Q9UQ80](http://www.uniprot.org/uniprot/Q9UQ80) | PA2G4 | 15 | 8 | MGVVEcAK |
| PKG1 | [P00558](http://www.uniprot.org/uniprot/P00558) | PGK1 | 11 | 7 | NNQITNNQR |
| RAB1A | [P62821](https://www.uniprot.org/uniprotkb/P62821/entry) | Rab1a | 7 | 5 | mGPGATAGGAEK |
| RPA1 | [P27694](http://www.uniprot.org/uniprot/P27694) | RPA1 | 5 | 4 | VSDFGGR |
| RPL10A | [P62906](http://www.uniprot.org/uniprot/P62906) | RPL10A | 7 | 2 | KKYDAFLASESL |
| RPSA | [P08865](http://www.uniprot.org/uniprot/P08865) | RPSA | 10 | 2 | SSRNTGQRAV |
| STAT1 | [P42224](http://www.uniprot.org/uniprot/P42224) | STAT1 | 5 | 2 | LTQNALINDELVE |
| Vimentin | [P20152](https://www.uniprot.org/uniprotkb/P20152/entry) | Vim | 63 | 19 | QVDQLTNDKAR |
| α-ACTN4 | [O43707](http://www.uniprot.org/uniprot/O43707) | ACTN4 | 5 | 2 | DAEDIVNTARPDE |


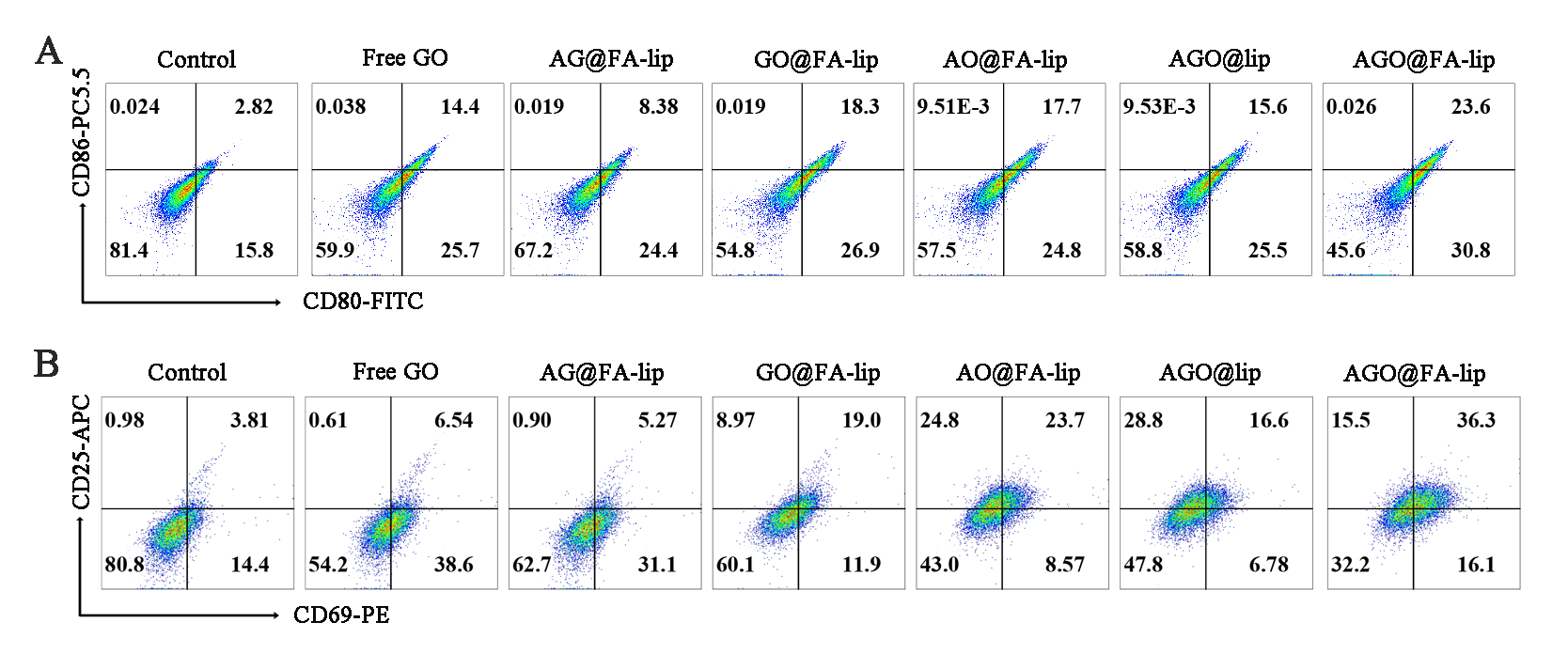


Figure S7. A. Flow cytometry detection of CD80/CD86 expression on BMDCs after co-incubation with captured antigens. B. Flow cytometry detection of T cell CD25/CD69 expression after 24-hour co-culture with DCs. * *p* < 0.05, ***p* < 0.01, ****p* < 0.001, *****p* < 0.0001.


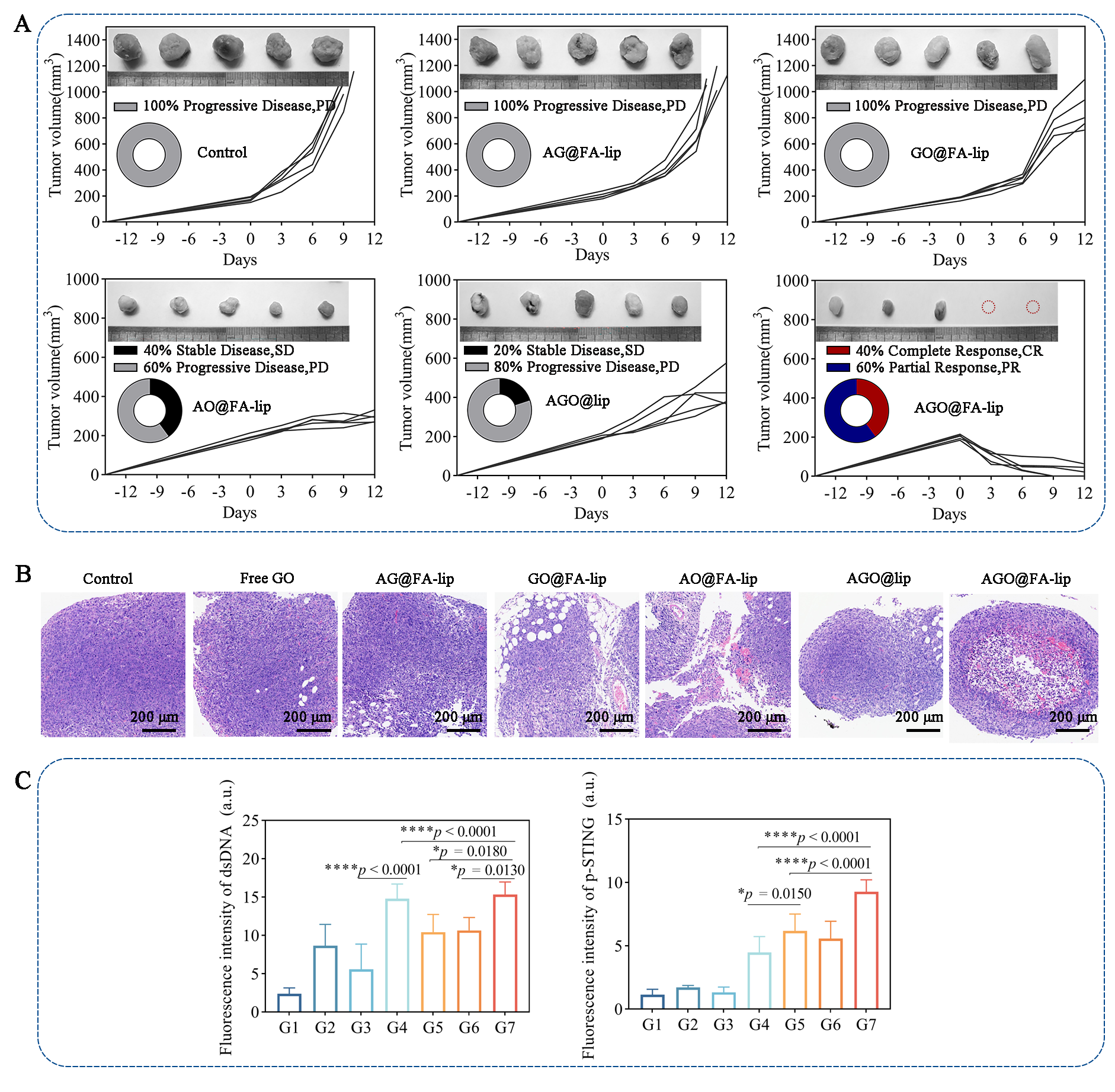


Figure S8. A. Tumor volume changes, tumor anatomy images, and RECIST (Response Evaluation Criteria in Solid Tumors) assessment results of subcutaneous tumor-bearing mice after intravenous administration of different nanoprobes (n = 5). B. H&E staining of intraperitoneal metastasis tumors after intraperitoneal injection of different nanoprobes. Scale bar 200 μm. C. Statistical analysis of the average fluorescence intensity of dsDNA and p-STING in subcutaneous transplanted tumor tissues after treatment with different nanoprobes (n = 3). * *p* < 0.05 , ***p* < 0.01, ****p* < 0.001, *****p* < 0.0001.


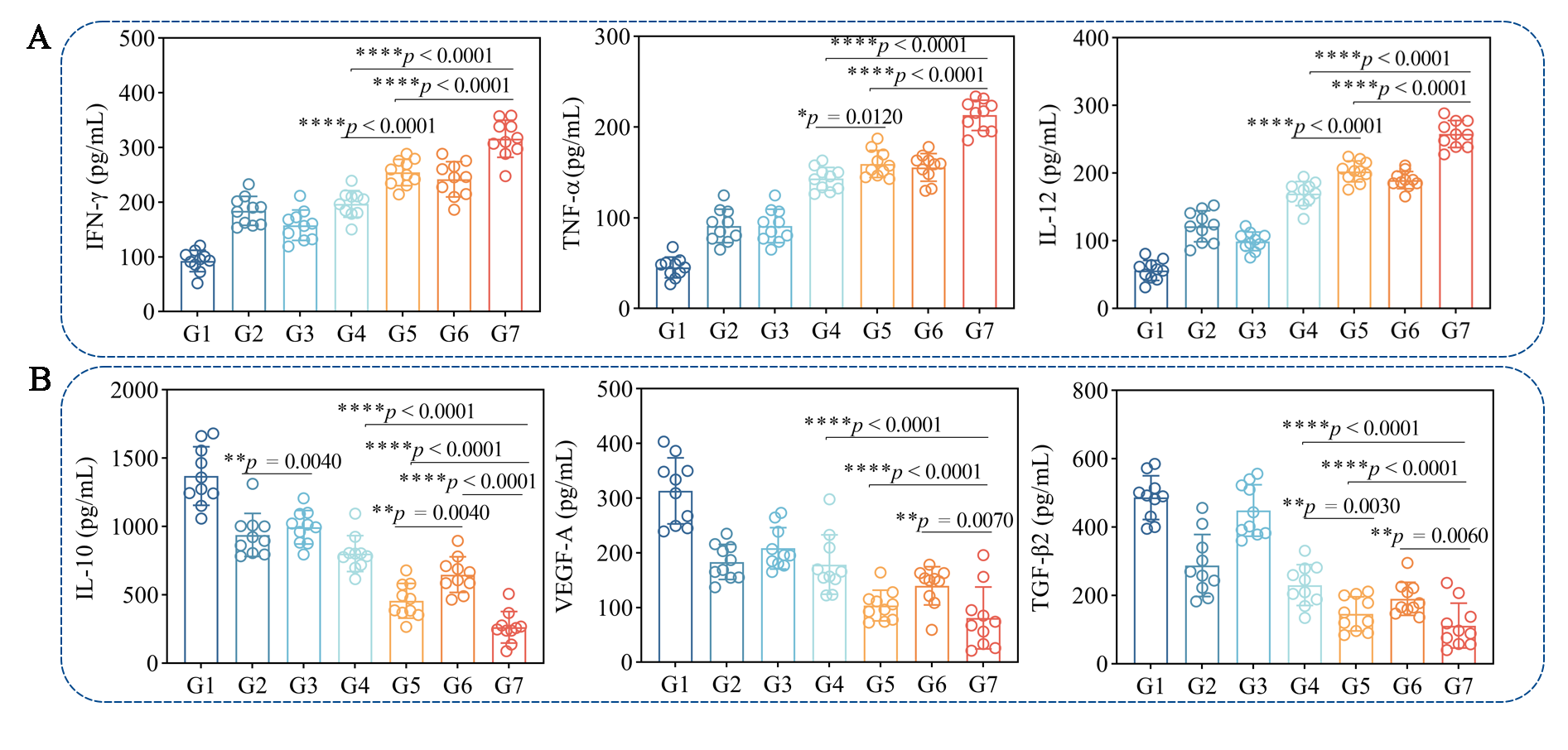


Figure S9. A. IL-12, IFN-γ and TNF-α in the serum of mice with subcutaneously transplanted tumors on the 7th day of treatment (n = 10). B. IL-10, VEGF-A and TGF-β2 in ascites of mice with intraperitoneal metastasis tumors on day 14 of treatment (n = 10). * *p* < 0.05 , ***p* < 0.01, ****p* < 0.001, *****p* < 0.0001.


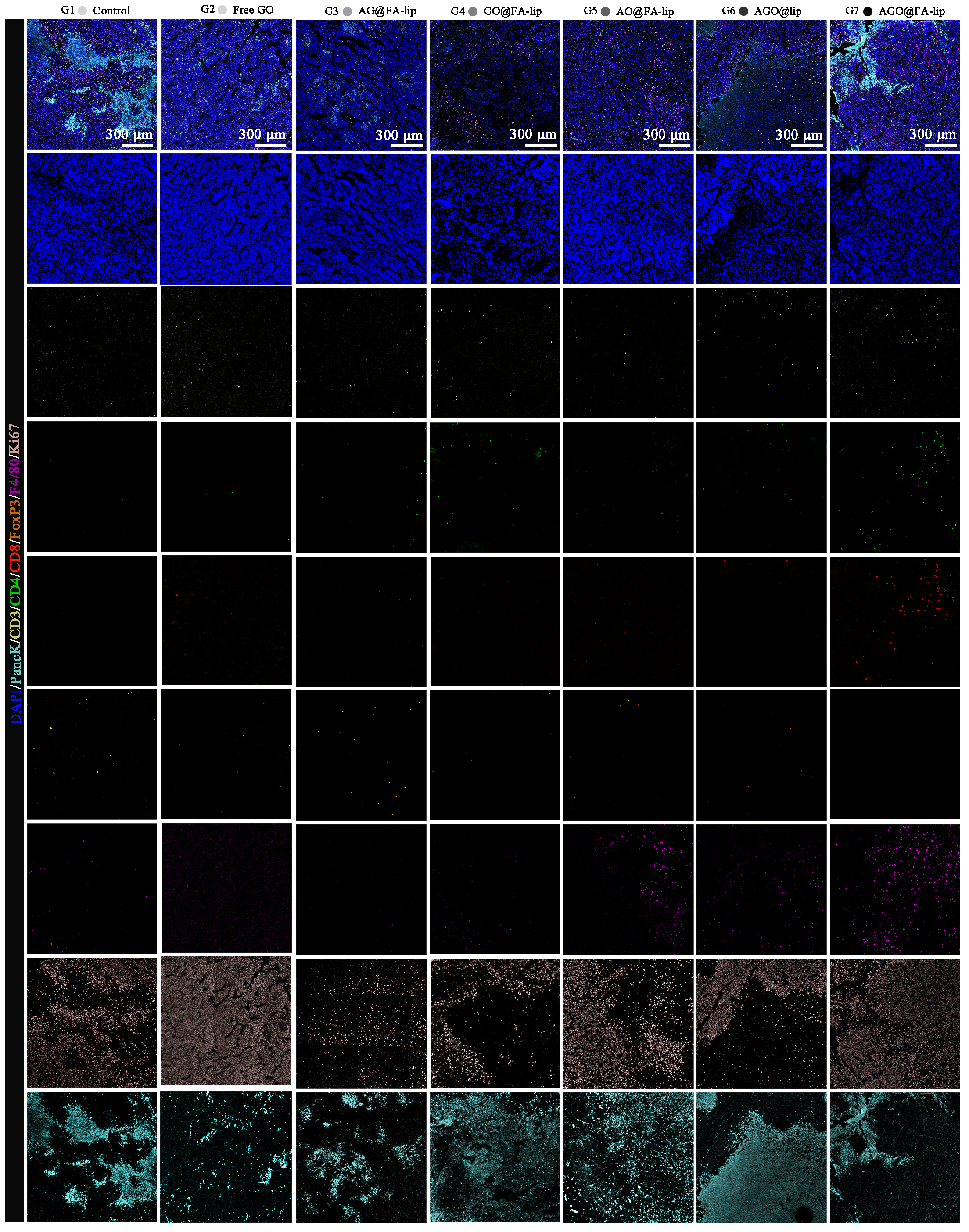


Figure S10. mIHC of CD3^+^ T cells, CD4^+^ T cells, CD8^+^ T cells, FoxP3^+^ T cells, F4/80 ^+^ macrophages, Ki67and Panck of the subcutaneous tumor-bearing mice after intravenous administration of different nanoprobes. Scale bar 300 μm. * *p* < 0.05, ***p* < 0.01, ****p* < 0.001, *****p* < 0.0001.

Table S5. Statistical analysis of immune cell counts in mIHC detection of tumor tissues from each group of mice

| Immune cell | G1 | G2 | G3 | G4 | G5 | G6 | G7 |
| --- | --- | --- | --- | --- | --- | --- | --- |
| CD3 | 120.33±40.07 | 362.70±29.85 | 550.33±83.01 | 831.33±76.00 | 1482.00±205.26 | 1122.67±222.53 | 1932.33±254.79*** |
| CD4 | 80.00±17.58 | 215.31±15.02 | 113.33±22.05 | 470.00±35.03 | 756.67±132.58 | 518.33±135.59 | 998.67±218.31**** |
| CD8 | 57.00±9.17 | 86.78±11.32 | 129.33±29.67 | 244.67±26.50 | 539.00±61.02 | 255±44.58 | 721.15±65.23**** |
| FoxP3 | 529.33±97.83 | 271.07±24.11 | 255.00±44.58 | 179.33±19.73 | 110.00±16.52 | 203.00±37.47 | 73.00±22.54*** |
| F4/80 | 329.00±36.66 | 694.15±73.49 | 516.67±164.63 | 1068.33±126.30 | 1358.67±270.53 | 1268.00±285.08 | 1929.67±296.70**** |

Data are shown as mean ± SD (n = 3). * *p* < 0.05, ***p* < 0.01, ****p* < 0.001, *****p* < 0.0001, ns *p* > 0.05.


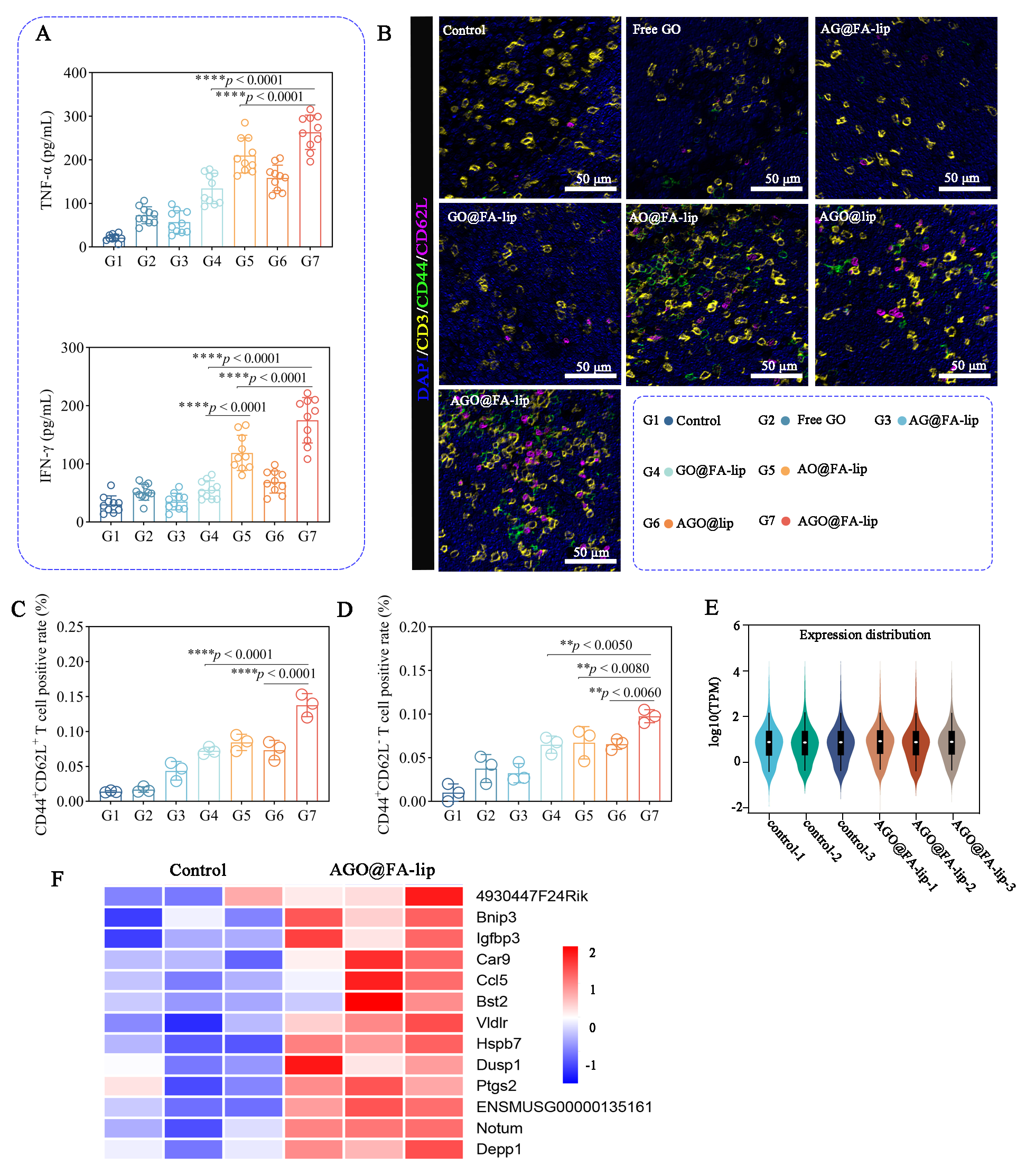


Figure S11. A. Concentrations of TNF-α and IFN-γ in tumors of mice after receiving different nanoprobes intravenous treatments measured by ELISA (n = 10). B. mIHC of CD3^+^CD44^+^CD62L^+^ T cells and CD3^+^CD44^+^CD62L^-^ T cells of tumor-draining lymph nodes. Scale bar 50 μm. C. Statistical analysis of CD44^+^CD62L^+^ T cell positivity in tumor-draining lymph nodes by mIHC (n = 3). D. Statistical analysis of CD44^+^CD62L^-^ T cell positivity in tumor-draining lymph nodes by mIHC (n = 3). E. Quantitative analysis of overall gene and transcript expression levels in tumor tissues from ID8 ovarian tumor-bearing mice post-treatment. F. Heatmaps showing upregulation of GO pathway-related genes in tumor tissues after treatment. * *p* < 0.05, ***p* < 0.01, ****p* < 0.001, *****p* < 0.0001, ns *p* > 0.05.


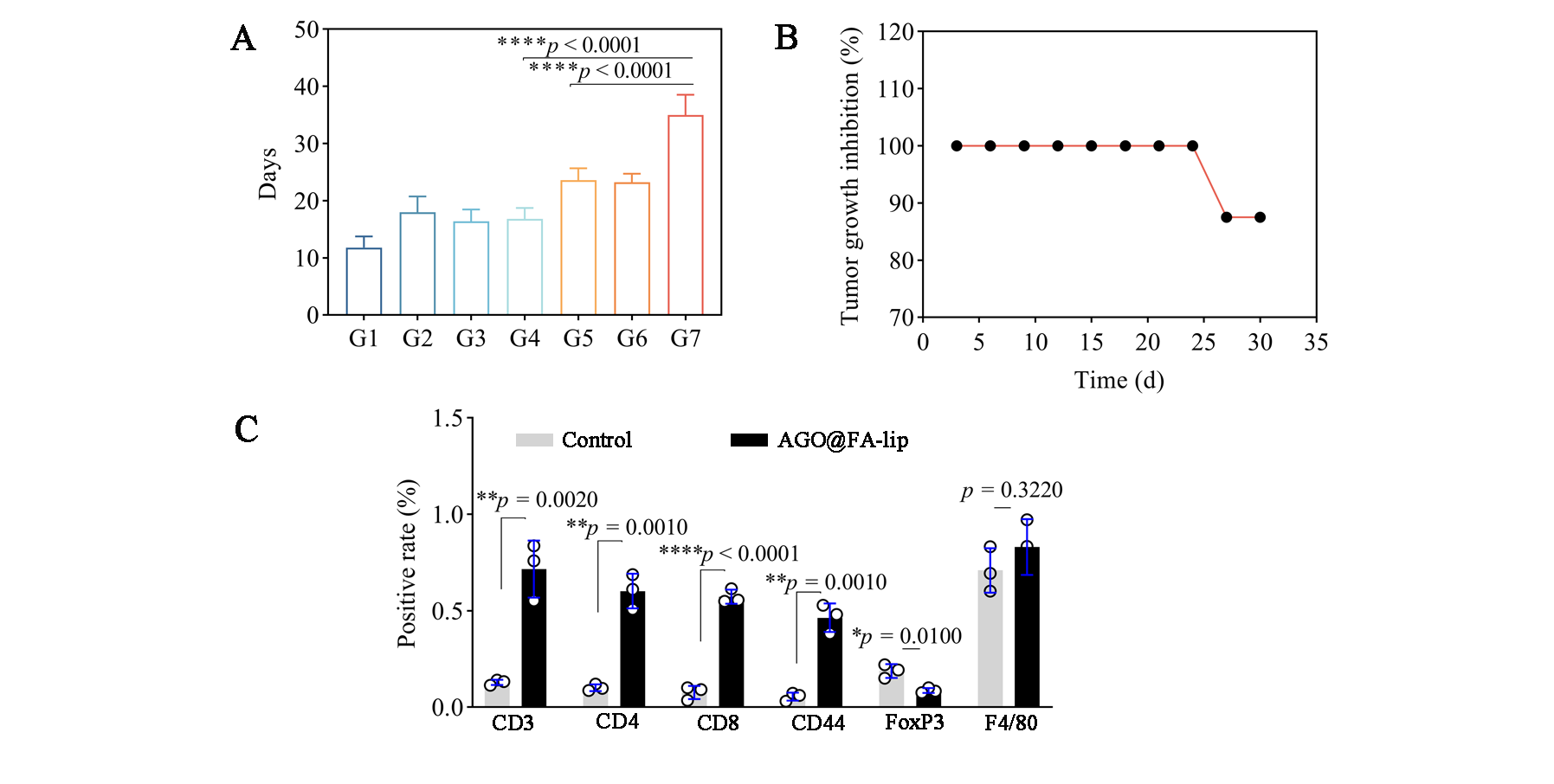


Figure S12. A. Time required for distant tumor volume to reach 500 mm³ in mice with distant tumors after treatment of primary tumors in each group (n = 5). B. Statistical analysis of immune cell positivity in distant tumors by mIHC in the control group and AGO@FA-lip group (n = 3). * *p* < 0.05, ***p* < 0.01, ****p* < 0.001, *****p* < 0.0001.


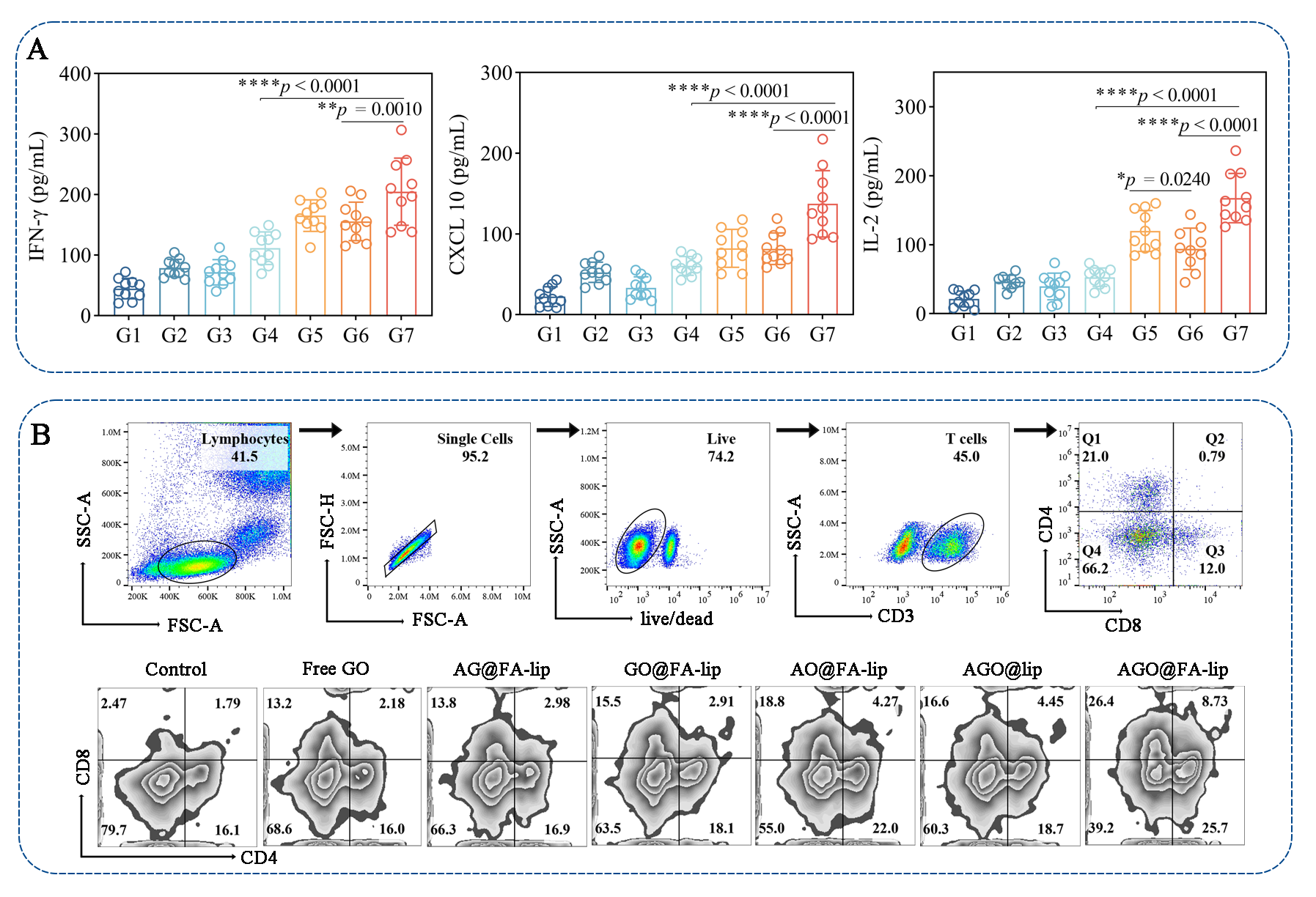


Figure S13. A. Concentrations of IFN-γ, CXCL10 and IL-2 in distant tumors measured by ELISA (n = 10). B. FCM detection of CD3^+^CD4^+^ T cells and CD3^+^CD8^+^ T cells in the spleens of mice with distant tumors after treatment. * *p* < 0.05, ***p* < 0.01, ****p* < 0.001, *****p* < 0.0001.
